# The mesophilic archaeon *Methanosarcina acetivorans* counteracts uracil in DNA with multiple enzymes: EndoQ, ExoIII, and UDG

**DOI:** 10.1038/s41598-018-34000-x

**Published:** 2018-10-25

**Authors:** Miyako Shiraishi, Sonoko Ishino, Matthew Heffernan, Isaac Cann, Yoshizumi Ishino

**Affiliations:** 1 Department of Bioscience and Biotechnology, Graduate School of Bioresource and Bioenvironmental Sciences, Kyushu University, 6-10-1 Hakozaki, Higashiku, Fukuoka, 812–8581 Japan; 2 Department of Animal Science, University of Illinois at Urbana-Champaign, Urbana, Illinois USA; 3 Department of Microbiology, University of Illinois at Urbana-Champaign, Urbana, Illinois USA; 4 Institute for Universal Biology, University of Illinois at Urbana-Champaign, Urbana, Illinois USA; 5 Carl R. Woese Institute for Genomic Biology, University of Illinois at Urbana-Champaign, Urbana, Illinois USA; 6 Present Address: Department of Materials Engineering Science, Graduate School of Engineering Science, Osaka University, 1–3 Machikaneyamacho, Toyonaka, Osaka 560–0043 Japan

## Abstract

Cytosine deamination into uracil is one of the most prevalent and pro-mutagenic forms of damage to DNA. Base excision repair is a well-known process of uracil removal in DNA, which is achieved by uracil DNA glycosylase (UDG) that is found in all three domains of life. However, other strategies for uracil removal seem to have been evolved in Archaea. Exonuclease III (ExoIII) from the euryarchaeon *Methanothermobacter thermautotrophicus* has been described to exhibit endonuclease activity toward uracil-containing DNA. Another uracil-acting protein, endonuclease Q (EndoQ), was recently identified from the euryarchaeon *Pyrococcus furiosus*. Here, we describe the uracil-counteracting system in the mesophilic euryarchaeon *Methanosarcina acetivorans* through genomic sequence analyses and biochemical characterizations. Three enzymes, UDG, ExoIII, and EndoQ, from *M. acetivorans* exhibited uracil cleavage activities in DNA with a distinct range of substrate specificities *in vitro*, and the transcripts for these three enzymes were detected in the *M. acetivorans* cells. Thus, this organism appears to conduct uracil repair using at least three distinct pathways. Distribution of the homologs of these uracil-targeting proteins in Archaea showed that this tendency is not restricted to *M. acetivorans*, but is prevalent and diverse in most Archaea. This work further underscores the importance of uracil-removal systems to maintain genome integrity in Archaea, including ‘UDG lacking’ organisms.

## Introduction

Base deamination is one of the most commonly occurring forms of damage to DNA^1^. The hydrolytic deamination of cytosine, adenine, and guanine generates uracil, hypoxanthine, and xanthine, respectively, which are generated spontaneously under physiological conditions^1^. Deaminated base formation is promoted by both endogenous factors (e.g., metabolites) and exogenous stress (e.g., heat), and is accelerated in single-stranded DNA (ssDNA)^1–6^. If not removed, uracil, hypoxanthine, and xanthine will preferentially pair with adenine, cytosine, and thymine, respectively, during the next round of DNA replication, resulting in point mutations^1,4,7^. Cytosine is generally more susceptible to deamination than adenine and guanine^1^. Indeed, C:G to T:A mutations in bacterial genomes are usually associated with the deamination of cytosine; accordingly, bacterial genomes are considered to have evolved toward reducing the GC content so as to reduce the risk of such mutations^8,9^. These types of mutations are also frequently observed in the genomes of cancer patients^1,10^. Deaminated bases in DNA can also be generated by the misincorporation of dUMP, dIMP, and dXMP by DNA polymerase. Although these misincorporations are not mutagenic themselves, their accumulation is nevertheless cytotoxic^11–13^. Thus, to maintain genome stability, cells need to immediately remove mutagenic and cytotoxic deaminated bases—especially uracil—from DNA.

Deaminated bases are mainly removed from DNA through the base excision repair (BER) pathway in most organisms^14^. As the initial step, deaminated bases are recognized and released from DNA by a lesion-specific DNA glycosylase [e.g., uracil-DNA glycosylase (UDG) for uracil, 3-methyladenine DNA glycosylase (AlkA) for hypoxanthine], which hydrolyzes the *N*-glycosidic bond between the base and deoxyribose^15,16^. The DNA backbone with the resultant apurinic/apyrimidinic (AP) site is then incised by AP endonuclease and/or AP lyase, and the pathway is completed with subsequent DNA repair synthesis and ligation^14,17^.

UDG is found in all three domains of life—Bacteria, Archaea, and Eukarya—forming a single superfamily comprised of six distinct families based on amino acid sequence and structural similarities^18^. UDG family 1 is widely detected among Bacteria and Eukarya. As represented by the first UDG discovered in *Escherichia coli*, this protein family shows specificity for uracil in both ssDNA and dsDNA^19^. UDG family 2 is also widespread in Bacteria and Eukarya, but shows more limited conservation compared to family 1 and is highly specific for mismatches such as U:G and T:G (thymine can be formed by the deamination of 5-methylcytosine)^19,20^. UDG family 3 proteins are highly specific for uracil in ssDNA and are only found in higher eukaryotes^19^. UDG family 4, known as thermostable UDG, is widespread in the archaeal domain and is also found in some thermophilic bacteria. These enzymes can remove uracil from both ssDNA and dsDNA^20,21^. UDG family 5 is present in some bacteria and archaea, and has a distinctive catalytic mechanism from the other families, recognizing hypoxanthine and xanthine as well as uracil^20,22,23^. The family 6 proteins are found in all domains of life; however, only a few archaeal proteins have been characterized to date. Interestingly, the substrate specificity of this family is limited to hypoxanthine and xanthine and does not extend to uracil^24^. Given the increased mutagenesis through gene inactivation and its high evolutionary conservation, UDG has been considered as the prime repair enzyme for uracil in DNA in many organisms^25–27^.

Nevertheless, several organisms exploit different uracil repair mechanisms besides the UDG system. For example, the thermophilic euryarchaeon *Methanothermobacter thermautotrophicus* lacks a functional UDG, and instead uses exonuclease III (ExoIII) for uracil repair. ExoIII belongs to one of the two major AP endonuclease families: ExoIII (also called Xth) and endonuclease IV (also called EndoIV or Nfo). ExoIII from *M. thermautotrophicus* (Mth212) can cleave the 5′-side of uracil in DNA in addition to the AP site and is considered the sole enzyme responsible for uracil repair in this organism^28^. As the major human AP endonuclease, ExoIII (also called APE1) has also been described to exhibit cleavage activity toward uracil, but is substantially less effective than Mth212^29^. This type of AP endonuclease-initiated repair is commonly known as nucleotide incision repair^30,31^.

Moreover, a novel enzyme involved in uracil repair was recently identified from the hyperthermophilic euryarchaeon *Pyrococcus furiosus*, designated endonuclease Q (EndoQ)^32^. Based on amino acid sequence homology, the putative EndoQ homologs are present in most of the Euryarchaeota (one of the major phyla of Archaea) and in some limited groups in Bacteria^32^. Similar endonuclease activity was identified from an EndoQ homolog detected in the gram-positive bacterium *Bacillus pumilus*^33^. This enzyme generates a nick immediately 5′ to uracil, and at the hypoxanthine, xanthine, and AP site in DNA^32,33^. Notably, the endonuclease activity toward uracil (and the AP site) is identical between EndoQ and ExoIII.

This apparent overlap in uracil repair mechanisms in a single organism motivated us to further characterize and explore the diversity of enzymes and their potential compensatory effects. When inspecting the distribution of uracil repair enzymes in Archaea, we noticed that the mesophilic euryarchaeon *Methanosarcina acetivorans* possesses at least four proteins that all potentially target uracil: two family-4 UDGs, ExoIII, and EndoQ, which were deduced from the amino acid sequence similarity. Here, we report the biochemical characterization of these four proteins found in *M. acetivorans*. We further provide an overview of the uracil-counteracting systems present in the archaeal domain in the context of their unique environments and evolutionary history. In particular, since most of the archaea identified to date are extremophiles, the ancestral organism of many archaeal mesophiles (including *M. acetivorans*) was most likely a thermophile living in an extreme environment, where organisms may be more susceptible to DNA damage, thereby requiring a more efficient repair system^34–37^.

## Results

### Preparation of MacEndoQ, MacExoIII, and MacUDG

**Figure 1:**
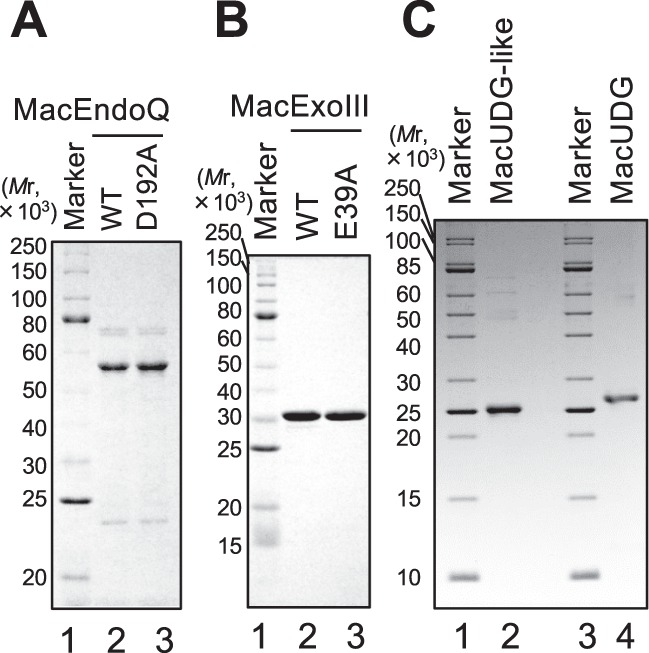
Preparation of recombinant proteins. Each purified protein (1 μg) was run on 12% (**A**,**B**) or 15% (**C**) SDS-PAGE, followed by CBB staining. The molecular weights of the markers are shown on the left of the panels. (**A**) Lane 1, protein marker (NEB, P7703); lane 2, MacEndoQ^WT^ (MW: 54111.1); lane 3, MacEndoQ^D192A^ (MW: 54067.1). (**B**) Lane 1, protein marker (NEB, P7703); lane 2, MacExoIII^WT^ (MW: 32548.5); lane 3, MacExoIII^E39A^ (MW: 32490.4). (**C**) Lanes 1 and 3, protein marker (NEB, P7704); lane 2, MacUDG-like (MW: 25231.11); lane 4, MacUDG (MW: 25298).

The recombinant proteins used in this study were overproduced in *E. coli* and purified close to homogeneity. The protein bands appearing in the gels were consistent with the calculated molecular weights of each N-terminal His-tagged protein (Fig. 1). To confirm that the nuclease activities are intrinsic to the proteins of interest, inactive mutants MacExoIII^E39A^ and MacEndoQ^D192A^ were prepared by introducing site-specific mutations into the active site predicted by sequence alignments with each family protein. The mutation E39A in MacExoIII was generated in accordance with a highly conserved residue among ExoIII homologs because of its involvement in metal binding that is essential for nuclease activity (Supplementary Fig. S1)^38–40^. The mutation D192A in MacEndoQ was generated in accordance with the essential residue for activity in *P. furiosus*^32^. The family-4 UDGs are the most prevalent UDGs in the archaeal domain and have been characterized as glycosylases specific for deoxyuridine (dU)^21^. Two putative family-4 UDGs, MA_RS11760 and MA_RS18745, were found in the *M. acetivorans* genome in the National Center for Biotechnology Information (NCBI) database. MA_RS18745 aligned better with the other characterized family-4 UDGs in Archaea, including the two catalytic motifs (Supplementary Fig. S2), and was therefore designated MacUDG. MA_RS11760 was found to deviate in the two catalytic motifs (Supplementary Fig. S2), and was therefore designated MacUDG-like.

### MacEndoQ acts on uracil, hypoxanthine, and the AP site in DNA

**Figure 2:**
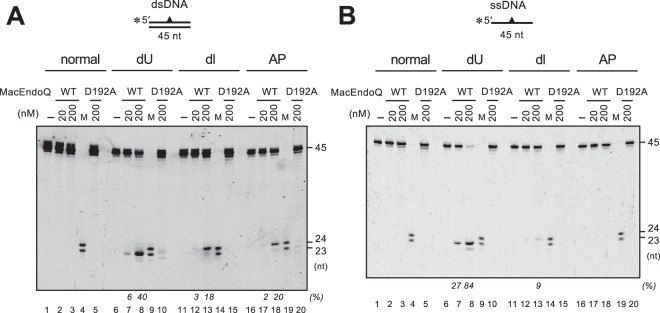
MacEndoQ exhibits dU, dI, and AP endonuclease activities. 5′-Cy5-labeled blunt-ended dsDNA (**A**) or ssDNA (**B**) containing dU (A & B; lanes 6–8, 10), dI (**A**,**B**; lanes 11–13, 15), or the AP site (**A**,**B**; lanes 16–18, 20) were incubated without protein (**A**,**B**; lanes 1, 6, 11, 16) or with 20/200 nM of MacEndoQ^WT^ (**A**,**B**; lanes 2, 3, 7, 8, 12, 13, 17, 18) or MacEndoQ^D192A^ (**A**,**B**; 200 nM; lanes 5, 10, 15, 20) at 37 °C for 1 h. M, DNA marker (**A**,**B**; lanes 4, 9, 14, 19). Cleavage products were separated by 8 M urea-12% PAGE. dU, dI and AP sites are at 24-, 25- and 25-nt from 5′-end, respectively. Fractions (%) of cleaved products in the total DNA bands per lane are indicated at the bottom of the gels in italic letters. The cropped gels are used in the figure, and the full-length gels are presented in Supplementary Fig. S8.

To characterize the activity of MacEndoQ, DNA cleavage assays were conducted using normal or single damaged base-containing DNA. As shown in Fig. 2A, MacEndoQ^WT^ recognized and cleaved the DNA containing dU, deoxyinosine (dI), and AP site, generating 23-, 24-, and 24-nucleotide (nt) fragments, respectively. In contrast, no product was detected in the case of no DNA damage or with the inactive mutant MacEndoQ^D192A^. The prepared MacEndoQ sample was also incubated with supercoiled plasmid DNA/circular ssDNA, and we did not observe any non-specific endonuclease contamination (Supplementary Fig. S3). The opposite strand of the damaged site was found intact (Supplementary Fig. S4). These results indicate that MacEndoQ incised the DNA backbone at the 5′-sides of the damaged sites. Similar cleavage pattern was observed when using ssDNA (Fig. 2B). The cleavage activity toward AP-ssDNA was not detected due to the low cleavage efficiency compared to that toward dsDNA (compare Fig. 2A,B, lane 18) under this condition. Such decreased cleavage efficiency towards AP-ssDNA has also been previously described for other characterized EndoQ homologs^32,33^. These findings suggest that MacEndoQ possesses endonuclease activity with substrate specificity as observed for EndoQ of the hyperthermophilic order *Thermococcales*, and the bacterial EndoQ from *B. pumilus*^32,33^. Thus, MacEndoQ appears to be involved in the repair of dU, dI, and AP sites. This finding further suggests that other putative EndoQ homologs in Euryarchaeota are most likely to be functional, taking into consideration the high conservation in protein sequences and phyletic analysis of this protein^33^.

### MacExoIII shows dU endonuclease activity as well as AP endonuclease activity

**Figure 3:**
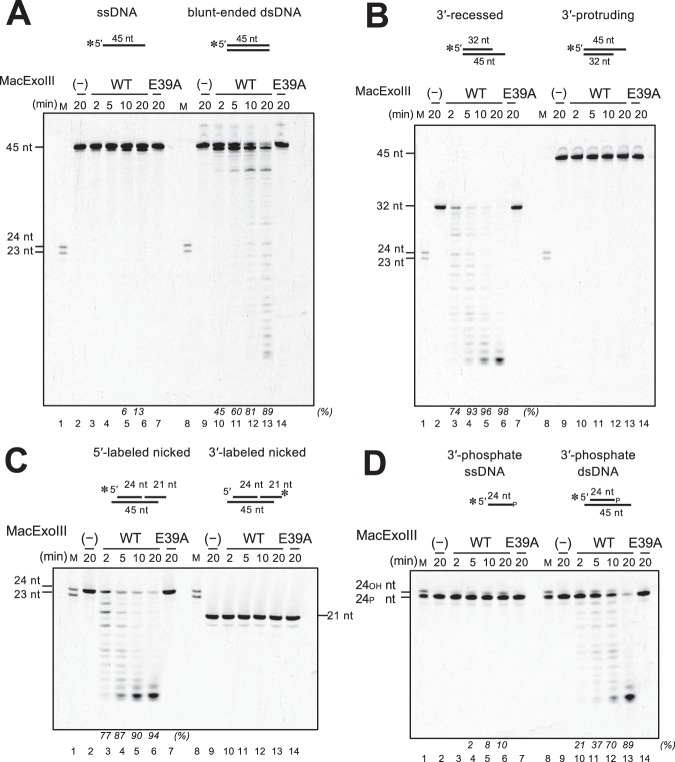
MacExoIII displays the properties conserved in ExoIII family members. DNA substrates were incubated with or without 1 nM MacExoIII^WT^ or 1 nM MacExoIII^E39A^ at 37 °C for the indicated times (2, 5, 10, 20 min). Cleavage products were separated by 8 M urea-15% PAGE. M, DNA marker (lanes 1 and 8). (−), no enzyme control (lanes 2 and 9). (**A**) 5′-Cy5-labeled ssDNA (lanes 1–7) or dsDNA (lanes 8–14). (**B**) 5′-Cy5-labeled 3′-protruding (lanes 1–7) or 5′-protruding dsDNA (lanes 8–14). (**C**) 5′-Cy5-labeled (lanes 1–7) or 3′-Cy5-labeled (lanes 8–14) 6-nt 3′-overhang nicked DNA. (**D**) 5′-Cy5-labeled ssDNA (lanes 1–7) or dsDNA (lanes 8–14) with 3′-phosphate termini. Amounts of cleaved DNA are determined by subtracting amounts of uncleaved substrates (full-length bands) from amounts of the initial substrates on the control lanes, and fractions (%) of cleaved DNA are indicated at the bottom of the gels in italic letters. The cropped gels are used in the figure, and the full-length gels are presented in Supplementary Fig. S9.

**Figure 4:**
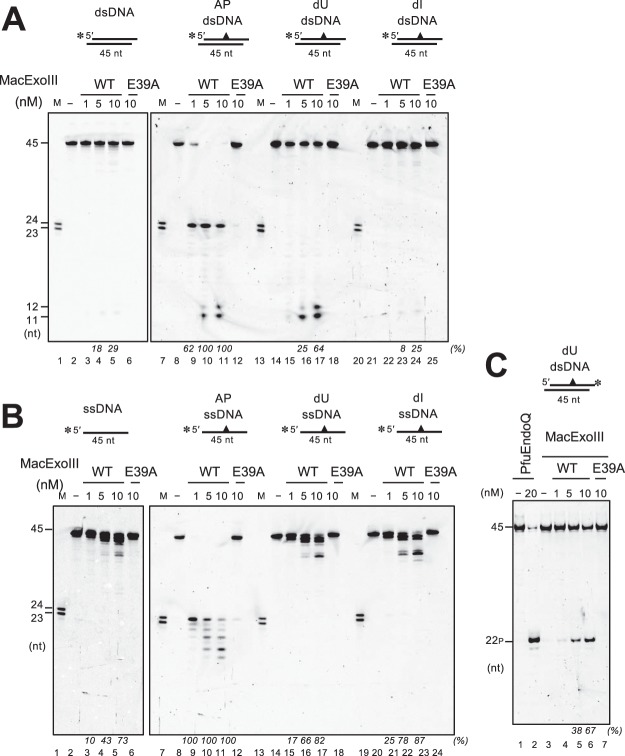
MacExoIII has endonuclease activity for dU-dsDNA and AP-DNA. 5′-Cy5-labeled 6-nt 3′-overhang DNA (**A**; lanes 2–6, 8–12, 14–18, 20–24) or 5′-Cy5-labeled ssDNA (**B**; lanes 2–6, 8–12, 14–18, 20–24) were incubated without protein (**A**,**B**; lanes 2, 8, 13, 20) or with MacExoIII^WT^ (1, 5, 10 nM) (**A**,**B**; lanes 3–5, 9–11, 15–17, 21–23) or 10 nM MacExoIII^E39A^ (**A**,**B**; lanes 6, 12, 18, 24) at 37 °C for 10 min. DNA substrates, normal DNA (**A**,**B**; lanes 2–6) or damaged DNA (**A**,**B**: AP site, lanes 8–12; dU, lanes 14–18; dI, lanes 20–24). M, DNA marker (**A**,**B**; lanes 1, 7, 13). 3′-Cy5-labeled 6-nt 3′-overhang DNA containing dU (**C**; lanes 1–7) incubated without (**C**; lanes 1 and 3) or with MacExoIII^WT^ (1, 5, 10 nM) (**C**; lanes 4–6), 10 nM MacExoIII^E39A^ (**C**; lane 7), or 20 nM PfuEndoQ (**C**; lane 2) at 37 °C for 10 min. Cleavage products were separated by 8 M urea-15% PAGE. The lengths of the fragments are indicated on the left sides of the gels. Amounts of cleaved DNA are determined by subtracting amounts of uncleaved substrates (full-length bands) from amounts of the initial substrates on the control lanes, and fractions (%) of cleaved DNA are indicated at the bottom of the gels in italic letters. The cropped gels are used in the figure, and the full-length gels are presented in Supplementary Fig. S10.

The ExoIII (Xth) protein family has been intensively investigated as 3′-5′ exonucleases and AP endonucleases, and the homologs show high evolutionary conservation. In some organisms, the 3′-phosphodiesterase activity of these proteins has also been reported^41,42^. We first examined whether MacExoIII also exhibits these typical characteristics of the ExoIII family proteins (Fig. 3). MacExoIII^WT^ digested blunt-ended dsDNA successively (Fig. 3A, lanes 9–14), while the ssDNA remained almost completely intact (Fig. 3A, lanes 2–7). Using 3′-recessed dsDNA, the substrate was digested in the same manner as blunt-ended dsDNA (Fig. 3B, lanes 2–7), whereas 3′-protruding ended dsDNA showed resistance to degradation (Fig. 3B, lanes 9–14), indicating that MacExoIII^WT^ exhibits dsDNA-specific exonuclease activity from the 3′ to 5′ direction. A nick site in dsDNA (Fig. 3C, lanes 2–7) and dsDNA with 3′-phosphate termini (Fig. 3D, lanes 9–14) were also susceptible to digestion by MacExoIII^WT^, indicating that MacExoIII can exhibit exonuclease activity from a nick and possesses 3′-phosphomonoesterase activity. These properties of exonuclease activity are conserved in the typical ExoIII family proteins. In addition, we investigated the endonuclease activity of MacExoIII using DNA with or without damage (AP site, dU, or dI) (Fig. 4). To prevent the 3′-5′ exonuclease activity at the ends of DNA, a 3′-protruding structure was used in this experiment. When AP site-containing DNA was used, 24-nt products and shorter fragments were detected with both ssDNA and dsDNA, suggesting that MacExoIII^WT^ cleaved the DNA backbone at the 5′-side of the AP site, followed by exonuclease digestion (Fig. 4A and B, lanes 8–12). The endonuclease activities were only detected on the damaged strands (Supplementary Fig. S5). These findings are also consistent with the classical property of ExoIII, as represented by the homologs from *E. coli*, *M. thermautotrophicus*, and humans^41–43^. Therefore, importantly, these data suggest a role of MacExoIII as an AP endonuclease in the BER pathway. The limited length of the product by MacExoIII^WT^ exonuclease was observed to be 11 nt. MacExoIII may require at least 11-nt DNA for binding.

Next, we investigated the endonuclease activity of MacExoIII towards dU and dI in DNA. Using ssDNA with a larger amount of MacExoIII, only unspecific cleavage from the end was observed, and MacExoIII^WT^ did not show any specific endonuclease activity on either dU-ssDNA (Fig. 4B, lanes 14–18) or dI-ss/dsDNA (Fig. 4A,B, lanes 21–25) under this condition. In addition, we did not detect nicked products with 5′-labeled dU-dsDNA, and only very short DNA fragments were obtained (Fig. 4A, lanes 14–18). Further attempts to detect the intermediate forms of the observed products with decreased reaction time or a lower protein concentration were unsuccessful (data not shown). However, interestingly, when DNA labeled on the opposite side (3′-end) was used, 22-nt products were detected, demonstrating the incision by MacExoIII at the site immediately 5′ to dU (Fig. 4C). Since MacExoIII exhibited 3′-5′ exonuclease activity in a processive manner (Figs. 3B,C), it is possible that nicked products of dU-dsDNA might not be detected. It must be noted that intact AP-dsDNA was completely digested with 5 nM MacExoIII, while dU-dsDNA was not (compare Fig. 4A lane 10 with lane 16), indicating a strong preference for the AP site over dU. This preference can also be explained by the fact that uracil recognition is restricted to dsDNA, but not ssDNA. Surprisingly, the subsequent exonuclease activity from the resulting nick was suppressed with AP-DNA compared to that for dU or normal DNA (compare Fig. 3 with Fig. 4A). To investigate the binding affinity of MacExoIII toward normal, AP-, and dU- containing DNA, we conducted the electrophoretic mobility shift assay (Supplementary Fig. S6). MacExoIII exhibited a stable protein-DNA complex in the presence of AP-site. In contrast, a protein-DNA complex was not detected in the case of dU-containing DNA, and only the degraded DNA fragments were observed. Considering that MacExoIII produced nicked DNA at a concentration of 5 nM against AP-containing DNA (Fig. 4 and Supplementary Fig. S5), it is suggested that MacExoIII binds persistently to the AP site after cleaving the strand.

### MacUDG is functional and may act with both MacExoIII and MacEndoQ in a single pathway

**Figure 5:**
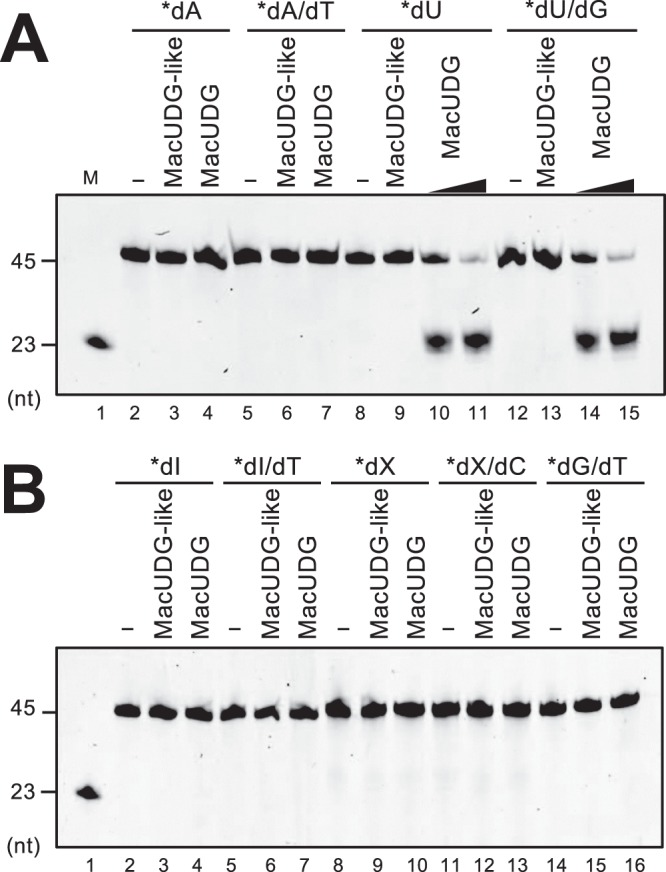
MacUDG exhibits glycosylase activity specific for uracil while MacUDG-like is inactive. 5′-Cy5-labeled ssDNA (**A**: lanes 2–4, 8–11; B: lanes 2–4, 8–10) or dsDNA (**A**: lanes 5–7, 12–15; **B**: lanes 5–7, 11–16) were incubated without (**A**: lanes 2, 5, 8, 12; **B** lanes 2, 5, 8, 11, 14) or with 1 μM MacUDG-like (**A**: lanes 3, 6, 9, 13; **B**: lanes 3, 6, 9, 12, 15), 1 nM MacUDG (**A**): lanes 10, 14), and 1 μM MacUDG (**A**: lanes 4, 7, 11, 15; **B**: lanes 4, 7, 10, 13,16) at 37 °C for 10 min. The products were treated with alkaline and heating, which cleaved DNA at AP site. DNA substrates are indicated at the top of the panels; dA, normal ssDNA; dA/dT, normal dsDNA; dU, dI, and dX, damaged ssDNA; dU/G, dI/T, and dX/C, damaged dsDNA; dG/dT, mismatched dsDNA. Asterisks represent Cy5-labeled strands. Cleavage products were separated by 8 M urea-10% PAGE. M, DNA marker (**A**,**B**, lane 1). The cropped gels are used in the figure, and the full-length gels are presented in Supplementary Fig. S11.

To investigate whether the two candidate proteins, MacUDG and MacUDG-like, have the ability to release damaged bases from DNA, enzymatic assays were performed using normal or damaged DNA. As shown in Fig. 5, MacUDG exhibited glycosylase activity towards uracil in DNA, but no activity was detected when using hypoxanthine, xanthine, and G/T mismatch-containing DNA. This result agrees with the common uracil-specific glycosylase activities detected in the family-4 archaeal UDGs^44–46^. By contrast, MacUDG-like did not show any activity even at a concentration of 1 μM in the reaction mixture (Fig. 5), which may reflect the lack of some key catalytic residues (Supplemental Fig. S2). To investigate whether MacUDG and AP endonucleases MacExoIII/MacEndoQ act in a single pathway in *M. acetivorans*, we carried out *in vitro* experiments to test whether MacExoIII/MacEndoQ cleaves the substrate generated by MacUDG. The results using dU-containing DNA showed that both MacExoIII and MacEndoQ can exhibit endonuclease activities on the product after UDG removes uracil from DNA (Supplementary Fig. S7).

### All genes encoding MacExoIII, MacEndoQ, MacUDG, and MacUDG-like are expressed in *M. acetivorans*

**Figure 6:**
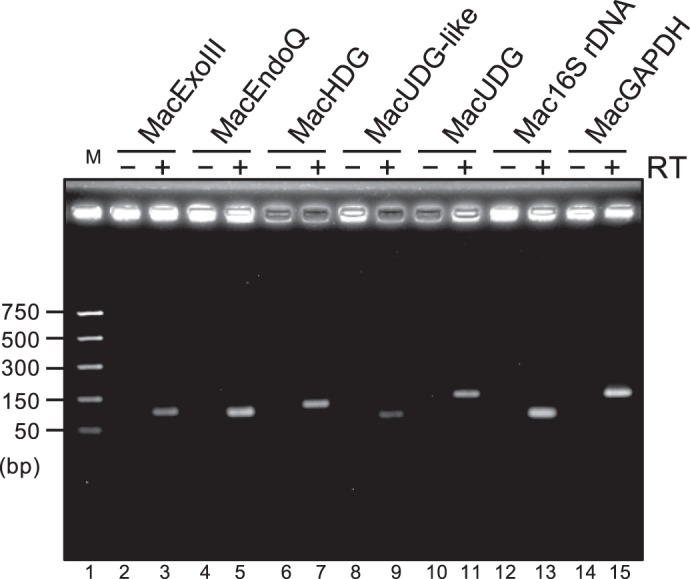
mRNAs of MacExoIII, MacEndoQ, MacUDGs detected by RT-PCR. cDNA was synthesized with (lanes 3, 5, 7, 9, 11, 13, and 15) or without (lanes 2, 4, 6, 8, 10, 12, and 14) reverse transcriptase using sequence-specific primers for MacExoIII (lanes 2 and 3), MacEndoQ (lanes 3 and 4), MacHDG (lanes 5 and 6), MacUDG-like (lanes 8 and 9), MacUDG (lanes 10 and 11), Mac16SrDNA (lanes 12 and 13, positive control), and MacGAPDH (lanes 14 and 15, positive control). cDNAs were amplified by PCR and the products were separated by 3% agarose gel electrophoresis, followed by ethidium bromide staining. M, DNA marker (NEB, #3234), lane 1.

To assess whether the genes encoding MacExoIII, MacEndoQ, MacUDG, and MacUDG-like are actually expressed in *M. acetivorans* cells, we conducted RT-PCR with or without RT enzyme using the gene-specific primers. Besides these four proteins, we also examined the expression of the genes for the family-6 UDG (designated as MacHDG)^24^. Expressions of the 16S rRNA gene and glyceraldehyde-3-phosphate dehydrogenase (GAPDH) were analyzed as positive controls of RT-PCR. As shown in Fig. 6, the mRNAs of MacExoIII, MacEndoQ, MacUDG, MacUDG-like, and MacHDG were detected as amplified DNA products when the RT enzyme was added. The sizes of the amplified DNA products were well-matched with the expected sizes (Fig. 6 and Supplementary Table S1). This result indicates that the mRNAs of each examined gene exist in *M. acetivorans* cells, suggesting that three different enzymes function in uracil repair. The gene for MacUDG-like was also expressed in the cells, although it does not have UDG activity. This protein might possess an as-yet-undiscovered cellular function.

### Distribution of enzymes involved in deaminated base repair in Archaea

**Figure 7:**
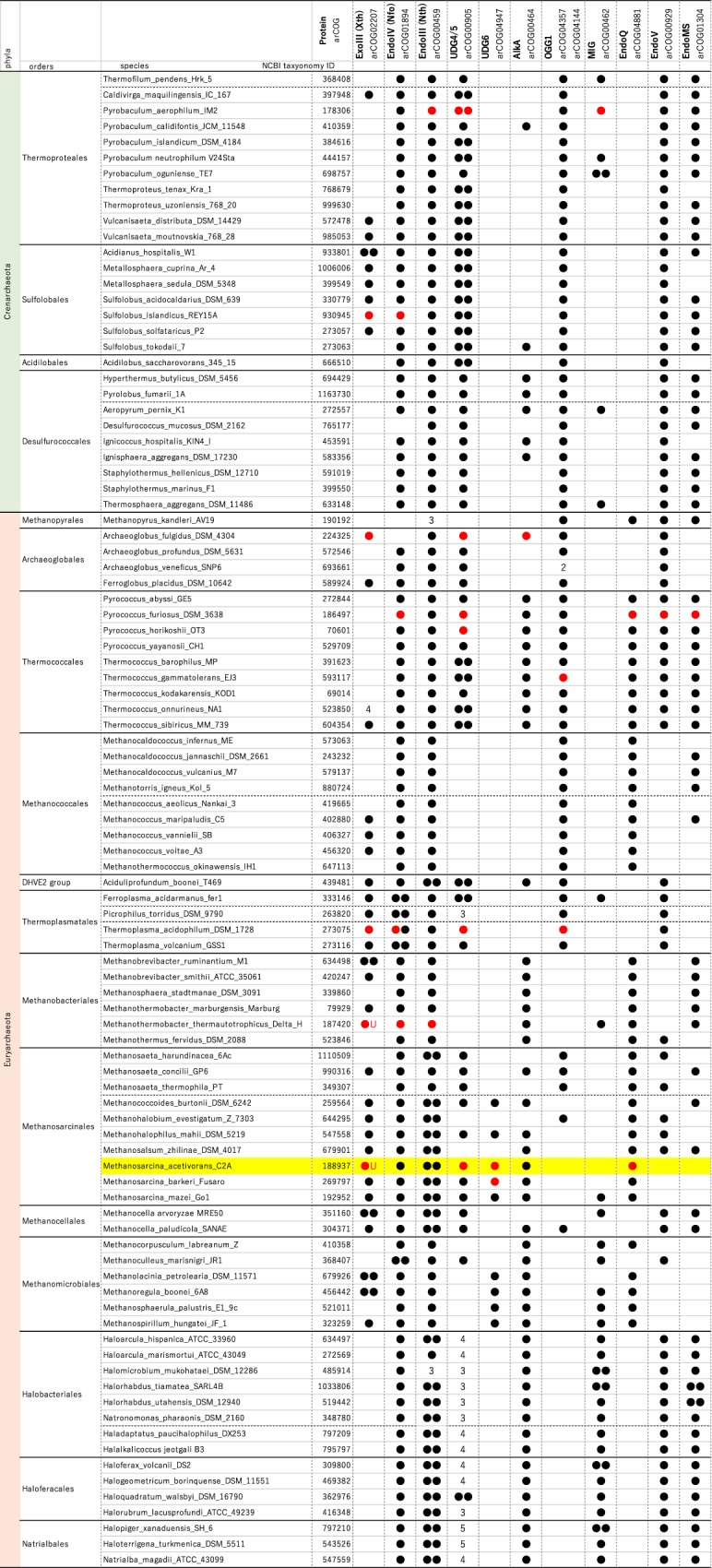
Distribution of DNA repair proteins involved in deaminated base repair. Red circles represent previously characterized homologs. Black circles represent the homologs at the amino acid sequence level. The numbers in the table represent the number of the homologs, if it is larger than 2. U indicates dU-endonuclease activity found in ExoIII.

To examine the distributions of the proteins for deaminated base repair in the domain Archaea, we performed a comprehensive search for homologs of 12 enzymes from 95 archaeal genome sequences: ExoIII, EndoIV, endonuclease III (EndoIII), family 4, 5, 6 UDGs, AlkA, 8-oxoguanine glycosylase (OGG1), U/G and T/G mismatch-specific glycosylase (MIG), EndoQ, endonuclease V (EndoV), and endonuclease MS (EndoMS). EndoIII from *Pyrobaculum aerophilum* has been described as an AP lyase and glycosylase for 5,6-dihydrothymine^47,48^. MIGs from *M. thermautotrophicus* and *P. aerophilum* have been described as glycosylases specific for mismatches, including U/G^49^. Archaeal EndoV incises the DNA backbone at the 1 nt 3′ to the hypoxanthine and is thought to be potentially involved in hypoxanthine repair^50,51^. EndoMS was recently identified as a mismatch-specific endonuclease from *P. furiosus*^52^, which cleaves both strands of hypoxanthine-containing DNA. Overall, this distribution analysis showed that several species have redundancy with respect to uracil repair proteins, suggesting that the archaeal domain has evolved a strong backup system (Fig. 7).

## Discussion

Removal of uracil to repair DNA damage is generally accomplished by ubiquitously conserved UDG proteins in all three domains of life. However, our *in vitro* data indicate that the mesophilic euryarchaeon *M. acetivorans* appears to have developed a different strategy to counteract uracil damage to DNA with at least three distinct enzymes: EndoQ, ExoIII, and family-4 UDG. We further confirmed the expression of the individual genes in *M. acetivorans*, and their substrate specificities agreed with those of previously reported archaeal homologs, suggesting the existence of overlapping uracil-counteracting systems in this single organism.

**Figure 8:**
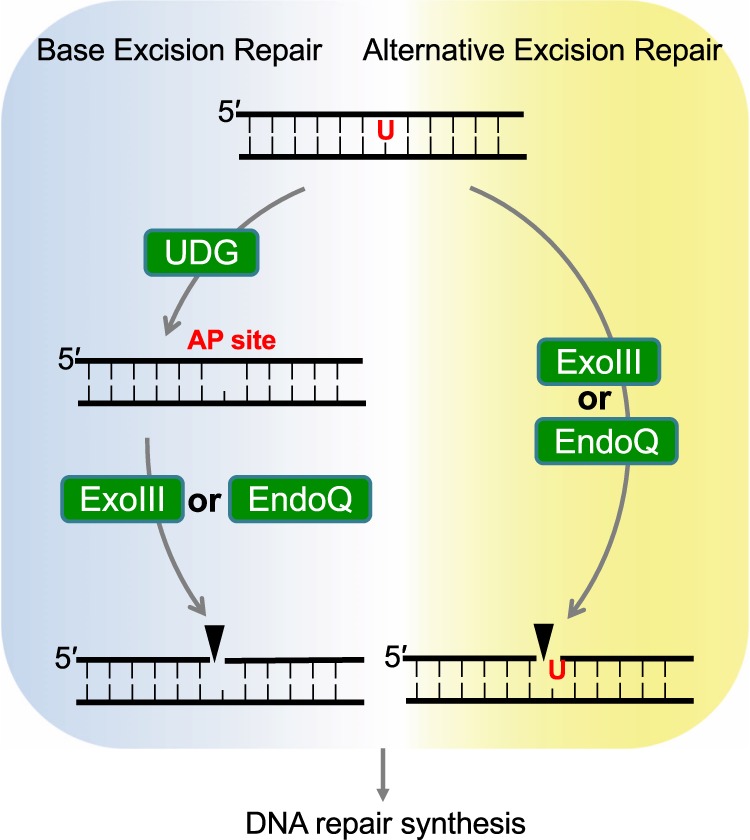
Schematic drawing of uracil repair pathway in *M. acetivorans*. Left pathway: Uracil in DNA is recognized by UDG to initiate the BER pathway. The resultant AP site is cleaved by either ExoIII or EndoQ. EndoIV in *M. acetivorans* may also act on AP site, although it is not drawn in this scheme. Right pathway: The other pathways of uracil repair are initiated by a direct DNA backbone cleavage by ExoIII or EndoQ (Alternative excision repair (AER) pathway). The resultant nicked DNA is subjected to the downstream repair pathway.

Based on data from a previous study demonstrating a correlation between bacterial genes and those present in the *M. acetivorans* genome^53^, we found that none of the proteins examined in this work (MacExoIII, MacEndoQ, MacUDG, and MacUDG-like) was acquired by horizontal gene transfer from bacteria, indicating that these proteins evolved in the archaeal domain and/or ancestor. We further revealed that (i) MacExoIII, MacEndoQ, and MacUDG all have enzymatic activity with uracil-specificity; (ii) their distributions across Archaea are not associated with each other; and (iii) there is a lack of sequence homologies in these proteins, indicating that the three proteins may not share a common ancestor (i.e., not derived by gene duplications). Therefore, we speculate that UDG, ExoIII, and EndoQ in *M. acetivorans* may comprise an individual repair pathway for uracil, each acting as a backup system for the other. Moreover, we found that MacExoIII and MacEndoQ may act on the MacUDG-catalyzed substrates (Supplemental Fig. S7), leading us to propose the potential uracil repair pathways in *M. acetivorans* (Fig. 8). Notably, MacExoIII, MacEndoQ, and MacUDG showed a unique range of substrate specificities and preferences. This may provide the driving force of the evolutionary stability underlying this redundancy^54^. Further studies are warranted to address which pathway is dominant or to determine the activation mechanisms of the respective pathway from this redundancy point of view.

A previous phylogenetic analysis of the UDG proteins raised a question of how the ‘UDG-lacking’ archaea can remove uracil from DNA^18^. A subsequent study proposed a potential answer by revisiting the properties of ExoIII from the ‘UDG-lacking’ organism *M. thermautotrophicus* to reveal its function as a dU endonuclease^28^. Here, we extend the understanding of uracil-counteracting systems in Archaea, including ‘UDG-lacking’ organisms. It appears that several archaeal ExoIIIs exhibit dU endonuclease activity based on analyses at the amino acid level. Such endonuclease activities towards oxidative DNA lesions have been described in various bacterial ExoIIIs such as *E. coli* and *Bacillus subtilis*^30,55^; however, since the uracil-recognition ability has only thus far been observed in Archaea and higher eukaryotes, we speculate that this recognition ability may have been acquired by the common ancestor of Archaea and Eukarya, as suggested previously^29^. Importantly, our results further suggest that EndoQ also functions in many archaea besides the *Thermococcales* and may play a key role in maintaining DNA free of uracil in ‘UDG-lacking’ organisms (Fig. 7 and earlier reports^32,33^). Thus, our findings highlight the substantial redundancy in uracil repair proteins in Archaea, raising a new question of what is the advantage of having multiple co-existing repair pathways. Moreover, several orders such as *Methanopyrales*, *Methanococcales*, and *Methanobacteriales* seem to have lost UDG proteins entirely; however, the conserved EndoQ may function as a uracil repair enzyme in these particular orders instead of UDG. MIGs appear to be dispersed, but may contribute to the removal of uracil in *Methanomicrobiales* together with EndoQ. As shown in Supplementary Fig. S1, many archaeal ExoIIIs bear the four key residues responsible for uracil recognition (Lys125, Asn153, Ser171, and Arg209 in Mth212^38^) and crucial residues for nuclease activity^39^, suggesting intrinsic dU endonuclease activity. This speculation is reinforced by the fact that human APE1, which has less overall homology, bears weak dU endonuclease activity^29^ even though the key Arg residue has been lost (Supplementary Fig. S1). The distribution map revealed some additional interesting facts. First, surprisingly, EndoIII was found to be ubiquitously distributed in Archaea. This high conservation implies a significant contribution of this protein in maintenance of the archaeal genome; however, this issue has been poorly investigated to date. Importantly, considering their high conservation, most of the archaeal organisms seem to conduct BER using EndoIV and family-4 UDGs, which is supported by previous studies^56–59^. We speculate that the archaeal BER pathway is regulated by the interplay of DNA glycosylases (UDG, MIG, AlkA, OGG1), AP lyase (EndoIII), and AP endonucleases (EndoIV, ExoIII).

Given that most archaea or their ancestral organisms are considered to be thermophiles^35^, we assume that an efficient backup system is required as a necessary survival tactic in the extreme environments where DNA would be more susceptible to base deamination. Furthermore, many organisms in Archaea appear to have gone through drastic habit changes in the course of evolution. For example, the ancestor of *M. acetivorans* had to cope with thermophilic to mesophilic temperatures, which would be considered an extreme environment. This can explain the relatively high diversity of otherwise conserved uracil-acting proteins, even among species classified in the same order such as *Methanosarcinales* or *Methamicrobiales* (Fig. 7). It must be noted that the same trend was also observed with respect to the conservation of hypoxanthine repair enzymes (Fig. 7).

Archaeal replicative DNA polymerases, members of the B and D families, have specific properties that result in stalling at the replication fork when encountering uracil in the template strand^60,61^; therefore, the association between uracil and DNA replication in Archaea is of particular interest. A previous study demonstrated inhibition of EndoQ and UDG activities by both family B and D DNA polymerases, suggesting potential interplay to prevent strand scission at the on-going replication folk^62^. To date, several archaeal family-4 UDGs have been reported to interact with proliferating cell nuclear antigen (PCNA). PCNA functions as a sliding clamp that tethers proteins such as DNA polymerase to DNA, thereby acting as a scaffold to facilitate the events on DNA. The interactions between PCNA and its interacting proteins are often achieved via the consensus peptide PCNA-interacting protein-box (PIP-box)^63^. Family-4 UDG and EndoIV from *P. furiosus* have been proposed to act together by interacting with PCNA^44,44^. This interaction appears to be well-conserved from archaeal UDGs to the human nuclear UDG (UNG2)^45,64,65^, indicating strong coordination with PCNA; however, MacUDG lacks the PIP-box found in *P. furiosus* (Supplementary Fig. S2). Moreover, to our knowledge, archaeal ExoIIIs have not been found to interact with PCNA and other PCNA-interacting proteins. In contrast, only MacEndoQ seems to interact via the PIP-box^33^. Although it remains possible that MacUDG and MacExoIII could interact with PCNA via an unidentified motif, the gene seems to have been selected among archaeal species during evolution, and likewise of the interacting partners. Since our study was focused on *in vitro* protein characterization, it is currently not clear how these proteins mediate the pathway or in what context they are activated. In future studies, revealing the functional connections of these uracil-acting proteins with DNA replication and repair-associated proteins may provide new clues into the development of cellular mechanisms for maintaining genome integrity.

## Materials and Methods

### Cloning of expression plasmids

The genes of MacExoIII [AAM05478.1, MA_RS10790 (MA2077)], MacEndoQ [AAM04083.1, MA_RS03380 (MA0641)], MacUDG-like [WP_052279161, MA_RS11760 (MA2265)], and MacUDG [AAM06949.1, MA_RS18745 (MA3593)] were amplified by PCR directly from *M. acetivorans* genomic DNA using gene-specific primers (Supplementary Table S1). Each amplified gene was digested with NdeI and NotI and ligated into the corresponding sites of the expression vector pET22–28TEV, which is a modified plasmid of pET21d (Novagen) from a thrombin recognition site to the TEV protease recognition site and from the kanamycin-resistant gene to the ampicillin-resistant gene. The resulting plasmids were designated pET-MA_RS10790^WT^, pET-MA_RS03380^WT^, pET-MA_RS11760, and pET-MA_RS18745. The expression plasmids for MacExoIII with the E39A mutation and for MacEndoQ with the D192A mutation were generated using the primers MA_RS10790-E39A and MA_RS03380-D192A (Supplementary Table S1) and the QuikChange Lightning Site-Directed Mutagenesis Kit (Agilent Technologies) according to the manufacturer’s instructions. The resulting plasmids were designated pET-MA_RS10790^E39A^ and pET- MA_RS03380^D192A^, respectively. The nucleotide sequences of the inserted regions of all plasmids were confirmed by sequencing.

### Protein purification of MacExoIII

MacExoIII^WT^ was overproduced in *E. coli* BL21 CodonPlus (DE3)-RIL (Agilent Technologies) cells carrying pET-MA_RS10790^WT^. The cells were grown with shaking in 4 L of Luria-Bertani (LB) medium, containing 100 µg/mL ampicillin and 34 µg/mL chloramphenicol at 37 °C until the optical density at 600 nm (OD_600_) reached 0.1. The inducer isopropyl-β-d−1-thiogalactopyranoside (IPTG) was added to a final concentration of 0.1 mM and the cells were further grown at 16 °C overnight. The cells were harvested by centrifugation and sonicated in 90 mL buffer A (20 mM sodium phosphate, pH 7.4, 0.5 M NaCl, 0.05% Tween 20, and 10% glycerol) containing 1 mM phenylmethylsulfonyl fluoride (PMSF). The soluble cell extracts were obtained by centrifugation. The soluble fraction was subjected to a 5-mL HisTrap FF Crude column (GE Healthcare) and eluted with a linear gradient of 30–300 mM imidazole in buffer A. Fractions containing MacExoIII^WT^ as observed on a 12% SDS-PAGE gel, were pooled and 5-fold-diluted with buffer B (50 mM Tris-HCl, pH 8.0, 0.5 mM DTT, 0.05% Tween20, and 10% glycerol). The diluted fraction was subjected to a 1-mL HiTrap Heparin HP column (GE Healthcare). The column was developed with a linear gradient of 0.1–0.8 M NaCl in buffer B. The eluted protein fractions were stored at −20 °C with 50% glycerol. The inactive mutant MacExoIII^E39A^ was purified from cells carrying pET-MA_RS10790^E39A^ in the same manner as described for MacExoIII^WT^, except that the protein was prepared from 1-L culture and subjected onto a 1-mL HisTrap HP column instead of a 5-mL HisTrap FF Crude column. The purities of the proteins were evaluated by 12% SDS-PAGE followed by Coomassie Brilliant Blue (CBB) staining. The protein concentrations were determined by measuring the absorbance at 280 nm. The theoretical molar extinction coefficients of N-terminal 6× His-tagged MacExoIII^WT^ and MacExoIII^E39A^ are both 48,485.

### Protein purification of MacEndoQ

MacEndoQ^WT^ was overproduced in *E. coli* BL21 CodonPlus (DE3)-RIPL (Agilent Technologies) cells carrying pET-MA_RS03380^WT^. The cells were grown with shaking in 1 L of LB medium containing 100 µg/mL ampicillin and 34 µg/mL chloramphenicol at 37 °C until the OD_600_ reached 0.4–0.5. IPTG was added to a final concentration of 0.1 mM, and the cells were further grown at 16 °C overnight. The cells were harvested by centrifugation and sonicated in 20 mL buffer A containing 1 mM PMSF and 30 mM imidazole. The soluble cell extracts were obtained by centrifugation (15 min, 11,000 × *g*, 4 °C). The soluble fraction was subjected to a 1-mL HisTrap HP column (GE Healthcare) and eluted with 300 mM imidazole in buffer A. The buffer of the eluted protein fractions was exchanged for 50 mM Tris-HCl, pH 8.0, 0.05% Tween 20, 10% glycerol, 0.2 M NaCl, using Illustra MicroSpin G-25 columns (GE Healthcare). MacEndoQ^D192A^ was prepared from the cells carrying pET-MA_RS03380^D192A^ in the same manner as described for MacEndoQ^WT^. The purified proteins were stored at −20 °C after the addition of 50% glycerol. The purity of the protein was evaluated by 12% SDS-PAGE followed by CBB staining. The protein concentration was calculated by measuring the absorbance at 280 nm. The theoretical molar extinction coefficients of N-terminal 6× His-tagged MacEndoQ^WT^ and MacEndoQ^D192A^ are both 48,860.

### Protein purification of MacUDG and MacUDG-like

MacUDG and MacUDG-like were overproduced in *E. coli* BL21 CodonPlus (DE3)-RIPL (Agilent Technologies) cells carrying the respective plasmids pET-MA_RS11760 and pET-MA_RS18745. The cells were grown with shaking in 1 L of LB medium, containing 50 µg/mL ampicillin and 34 µg/mL chloramphenicol at 37 °C until the OD_600_ reached 0.6 for MacUDG-like and 0.4 for MacUDG. IPTG was added to a final concentration of 0.1 mM, and the cells were further grown at 18 °C overnight for MacUDG-like and at 16 °C overnight for MacUDG. The proteins were prepared in nearly the same manner as described for MacExoIII. The eluted protein fractions were stored at −20 °C with 50% glycerol or at −80 °C. The purities of the proteins were evaluated by 15% SDS-PAGE followed by CBB staining. The protein concentrations were calculated by measuring the absorbance at 280 nm. The theoretical molar extinction coefficients of N-terminal 6× His-tagged MacUDG-like and MacUDG are 21,110 and 21,930, respectively.

### Oligonucleotides

The oligonucleotides were obtained from Sigma-Aldrich (Tokyo, Japan) and Integrated DNA Technologies (USA). The sequences are shown in Supplementary Table S1, and S2. The combinations of the oligonucleotides for structured substrates are shown in Supplementary Table S3. Annealing of oligonucleotides was conducted in TAM buffer (40 mM Tris-acetate, pH 7.8 and 0.5 mM Mg(CH_3_COO)_2_) with a gradually decreasing temperature. The primers for RT-PCR were designed using Primer3Plus (http://primer3plus.com/cgi-bin/dev/primer3plus.cgi).

### Nuclease activity assay

The cleavage reactions for MacExoIII were performed at 37 °C for various time periods in a 20-μL reaction mixture containing 50 mM Bis-Tris-HCl, pH 7.0, 1 mM DTT, 1 mM MnCl_2_, 0.01% Tween 20, 5 nM DNA, and various concentrations of MacExoIII as described in the figure legends. The cleavage reactions for MacEndoQ were performed at 37 °C for 1 h in a 20-μL reaction mixture containing 50 mM Tris-HCl, pH 8.0, 1 mM DTT, 1 mM MgCl_2_, 0.01% Tween 20, 5 nM DNA substrates, and various concentrations of MacEndoQ as described in the figure legends. The reactions were terminated with 20 μL of stop solution (98% deionized formamide, 10 mM EDTA, and 0.1% OrangeG). After incubation at 95 °C for 5 min, the samples were immediately placed on ice. The samples were separated by 8 M urea-12/15% PAGE in TBE buffer (89 mM Tris-borate and 2.5 mM EDTA). The gel image was visualized with a Typhoon TRIO+ image analyzer (GE Healthcare). Resulting band intensities were quantified with ImageQuant TL software (GE healthcare).

### Glycosylase activity assay

The cleavage reactions for MacUDG-like and MacUDG were performed at 37 °C for 10 min in a 20 μL reaction mixture containing 50 mM Tris-HCl, pH 8.0, 1 mM DTT, 1 mM EDTA, 0.1 μg/mL BSA, 5 nM DNA, and various concentrations of proteins as described in the figure legends. The backbone of the DNA with an AP site was then cleaved by adding 2 μL of 200 mM NaOH and heating at 95 °C for 5 min, followed by adding 2 μL of 200 mM HCl for neutralization. The substrates were denatured with 20 μL of the stop solution and incubated at 95 °C for 5 min, followed by rapid cooling on ice. The samples were separated by 8 M urea-10% PAGE in TBE buffer. The gel image was visualized with a Typhoon TRIO+ image analyzer (GE Healthcare).

### RT-PCR

*M. acetivorans* C2A cells were obtained from the Japan Collection of Microorganisms (JCM, RIKEN, Ibaraki, Japan). The cells were cultivated anaerobically at 37 °C in high-salt liquid medium^66^ containing 125 mM methanol. When the OD_600_ of the cells reached 0.4, total RNA was purified using the RNeasy Mini Kit (QIAGEN) and the RNase-Free DNase Set (QIAGEN) according to the manufacturer’s instructions. RNA concentrations were determined from the absorbance at 260 nm. Reverse transcription was performed with the total RNA using the reverse primers specific to the genes of interest (Supplementary Table S1) and the PrimeScript™ RT reagent Kit with gDNA Eraser (Takara), according to the manufacturer’s instructions. The synthesized cDNAs were detected by PCR using gene-specific primer pairs (Supplementary Table S1), followed by 3% agarose gel electrophoresis and ethidium bromide staining.

### Sequence analysis

Orthologs of ExoIII, EndoIV, EndoIII, UDG (family 4, 5, and 6), AlkA, OGG1, MIG, EndoQ, EndoV, and EndoMS in cultivable archaea (95 species) were retrieved by searching the Archaeal Clusters of Orthologous Genes (arCOG) database^67^ using the EggNOG 4.5.1 program^68^. One representative protein was selected for searching the ortholog groups (protein of interest, query, arCOG): ExoIII, AAM05478.1, arCOG02207; EndoIV, AAM06910.1, arCOG01894; EndoIII (Nth), AAL63095.1, arCOG00459; UDG4, AAM06949.1, arCOG00905; UDG5, AAL63408.1, arCOG00905; UDG6, AAM03908.1, arCOG04947; AlkA, AAM06922.1, arCOG00464; MIG, AAF37270.1, arCOG00462; OGG, AAB90876.1, arCOG04357 and arCOG04144; EndoQ, AAM04083.1, arCOG04881; EndoV, AAL81111.1, arCOG00929; EndoMS, AAL80136.1, arCOG01304. Multiple protein sequence alignment was performed with MUSTLE alignment software in Geneious 11.0.5 (http://www.geneious.com)^69^.

## Electronic supplementary material

Supplementary Information

## Data Availability

The authors declare that the data supporting the findings of this study are available within the paper and its supplementary information files or from the corresponding authors upon reasonable request.
