# Supplementary material for: The mesophilic archaeon Methanosarcina acetivorans counteracts uracil in DNA with multiple enzymes: EndoQ, ExoIII, and UDG
Source: Sci Rep. 2018 Oct 25;8:15791. doi: 10.1038/s41598-018-34000-x (PMC6202378; doi:10.1038/s41598-018-34000-x)
Supplement: Supplementary file 1 — Supplementary Information [file 41598_2018_34000_MOESM1_ESM.pdf]

Supplementary Information

The mesophilic archaeon *Methanosarcina acetivorans* counteracts uracil in DNA with multiple enzymes: EndoQ, ExoIII, and UDG

Miyako Shiraishi[1, 4, 5 #*], Sonoko Ishino[1], Matthew Heffernan[4], Isaac Cann[2, 3, 4, 5], and Yoshizumi Ishino[1, 4, 5*]

[1]Department of Bioscience and Biotechnology, Graduate School of Bioresource and Bioenvironmental Sciences, Kyushu University, 6-10-1 Hakozaki, Higashiku, Fukuoka 812-8581, Japan; [2]Department of Animal Science, [3]Department of Microbiology, [4]Institute for Universal Biology, and [5]Carl R. Woese Institute for Genomic Biology, University of Illinois at Urbana-Champaign, Urbana, Illinois, USA

#Present address: Department of Materials Engineering Science, Graduate School of Engineering Science, Osaka University, 1-3 Machikaneyamacho, Toyonaka, Osaka 560-0043, Japan

*Correspondence to Miyako Shiraishi (m.shiraishi@chem.es.osaka-u.ac.jp) or Yoshizumi Ishino (ishino@agr.kyushu-u.ac.jp)

Table of Contents

TableS1 ---------------------------------- S-1
Table S2 ---------------------------------- S-2
Table S3 --------------------------------- S-3
Figure S1 ---------------------------------- S-4
Figure S2 ---------------------------------- S-5
Figure S3 ---------------------------------- S-6
Figure S4 ---------------------------------- S-7
Figure S5 ---------------------------------- S-8
Figure S6 ---------------------------------- S-9
Figure S7 ---------------------------------- S-10
Figure S8 ---------------------------------- S-11
Figure S9 -------------------------------- S-12,13
Figure S10 -------------------------------- S-14,15
Figure S11 -------------------------------- S-16

Supplementary Table S1. Oligonucleotides used for gene cloning and RT-PCR.

Name	Sequence (5′-3′)	Target gene (Locus tag)	Usage	PCR products (bp)
MA_RS10790-F	GGG<u>CATATG</u>CCGGAAAATTATAACCTTA	MacExoIII (MA_RS10790)	Gene cloning	
MA_RS10790-R	CCC<u>GCGGCCGC</u>TCAAACCTGAAATTCGAGTTC			
MA_RS10790-E39A	TCGATGTCATCTGTGTCCAGG<u>C</u>AACCAAAGCCTCCCCGGAAAA			
MA_RS03380-F	CGC<u>CATATG</u>AAAGTCAATGCAGACCTCCATC	MacEndoQ (MA_RS03380)		
MA_RS03380-R	CGC<u>GCGGCCGC</u>TTAAAAATCGAAAAGGGATTTCTGCCC			
MA_RS03380-D192A	CTTACCTTCCTTACAAACTCCG<u>C</u>TGCCCATTCCCCTTACACCAAC			
MA_RS11760-F	CGCG<u>CATATG</u>AAGCCTGTTGAATACGTTAAATG	MacUDGlike (MA_RS11760)		
MA_RS11760-R	CCG<u>GCGGCCGC</u>TTACTTAATTAACTTCGCAGCTTC			
MA_RS18745-F	GGG<u>CATATG</u>AAGGAGCTAGAAATTGTGGC	MacUDG (MA_RS18745)		
MA_RS18745-R	GCG<u>GCGGCCGC</u>TTACTGCCATAGATCCCTGTTT			
MA_RS10790-RT-F	TGGGAATCGAGGAGTTTGAC	MacExoIII (MA_RS10790)	RT-PCR	101
MA_RS10790-RT-R	CTGAGAGGCTTTTCCGTTTG			
MA_RS03380-RT-F	CAGCGATTATGCAGACAGGA	MacEndoQ (MA_RS03380)		98
MA_RS03380-RT-R	ATTCCCTTGCCAGTTTGTTG			
MA_RS02415-RT-F	CGATTTCTGGAGGCTAGTCG	MacHDG (MA_RS02415)		118
MA_RS02415-RT-R	GGCTTTAAACACGTCCCAGA			
MA_RS11760-RT-F	TCCTGGATCACGGGTTCTAC	MacUDGlike (MA_RS11760)		76
MA_RS11760-RT-R	ACCGTATCTGCCGAAATCAC			
MA_RS18745-RT-F	AGAAGGCCAAAAGCAAGTGA	MacUDG (MA_RS18745)		143
MA_RS18745-RT-R	ACTCCCCGTTCCAGTTTTCT			
MA_RS04665-RT-F	GGGTCTAAAGGGTCCGTAGC	Mac16SrDNA (MA_RS04665)		80
MA_RS04665-RT-R	AGTATCCCCCGAAAGCCTAA			
MA_RS17460-RT-F	CTTGACTTCGTACGGGTGGT	MacGAPDH (MA_RS17460)		148
MA_RS17460-RT-R	TGATCGGGCCTTTCTTTATG			

Supplementary Table S2. Oligonucleotides used for protein activity assays

#	Name	Sequence (5′-3′)	Description
**1**	23N	CGAACTGCCTGGAATCCTGACGA	DNA marker
**2**	24N	CGAACTGCCTGGAATCCTGACGAC	DNA marker
**3**	24N-3Phos	CGAACTGCCTGGAATCCTGACGAC-Pho	3′-phosphate ssDNA
**4**	32N	CGAACTGCCTGGAATCCTGACGACATGTAGCG	
**5**	45N	CGAACTGCCTGGAATCCTGACGACATGTAGCGAACGATCACCTCA	ssDNA (normal)
**6**	45-U24	CGAACTGCCTGGAATCCTGACGA**[dU]**ATGTAGCGAACGATCACCTCA	dU-ssDNA
**7**	45-I25	CGAACTGCCTGGAATCCTGACGAC**[dI]**TGTAGCGAACGATCACCTCA	dI-ssDNA
**8**	45-AP25	CGAACTGCCTGGAATCCTGACGAC**[AP]**TGTAGCGAACGATCACCTCA	AP-ssDNA
**9**	45-G25	CGAACTGCCTGGAATCCTGACGAC**[G]**TGTAGCGAACGATCACCTCA	
**10**	45-X27	CGAACTGCCTGGAATCCTGACGACAT**[X]**TAGCGAACGATCACCTCA	dX-ssDNA
**11**	21N-5Phos	5-Phos-ATGTAGCGAACGATCACCTCA	
**12**	32R	CGCTACATGTCGTCAGGATTCCAGGCAGTTCG	
**13**	45R	TGAGGTGATCGTTCGCTACATGTCGTCAGGATTCCAGGCAGTTCG	Complementary oligo of 45N for blunt-ended dsDNA
**14**	45R-3-overhang	GATCGTTCGCTACATGTCGTCAGGATTCCAGGCAGTTCGCTAGCC	Complementary oligo of 45N for 6 nt-3′-overhang dsDNA

Supplementary Table S3. Preparation for dsDNA.

Name	Combination*
blunt-ended dsDNA (normal) or dA/dT	5 + 13
blunt-ended dU-dsDNA or dU/dG	6 + 13
blunt-ended dI-dsDNA or dI/dT	7 + 13
blunt-ended AP-dsDNA or AP/dT	8 + 13
blunt-ended G/T mismached DNA or dG/dT	9 + 13
blunt-ended X-dsDNA or dX/dT	10 + 13
3′-recessed dsDNA	4 + 13
5′-protruding dsDNA	4 + 12
nicked dsDNA	2 + 11 + 14
3′-phosphate dsDNA	3 + 14
6 nt 3′-overhang dsDNA (normal)	5 + 14
6 nt 3′-overhang dU-dsDNA	6 + 14
6 nt 3′-overhang dI-dsDNA	7 + 14
6 nt 3′-overhang AP-dsDNA	8 + 14

*The numbers on the combination column correspond to those in Supplementary table S2

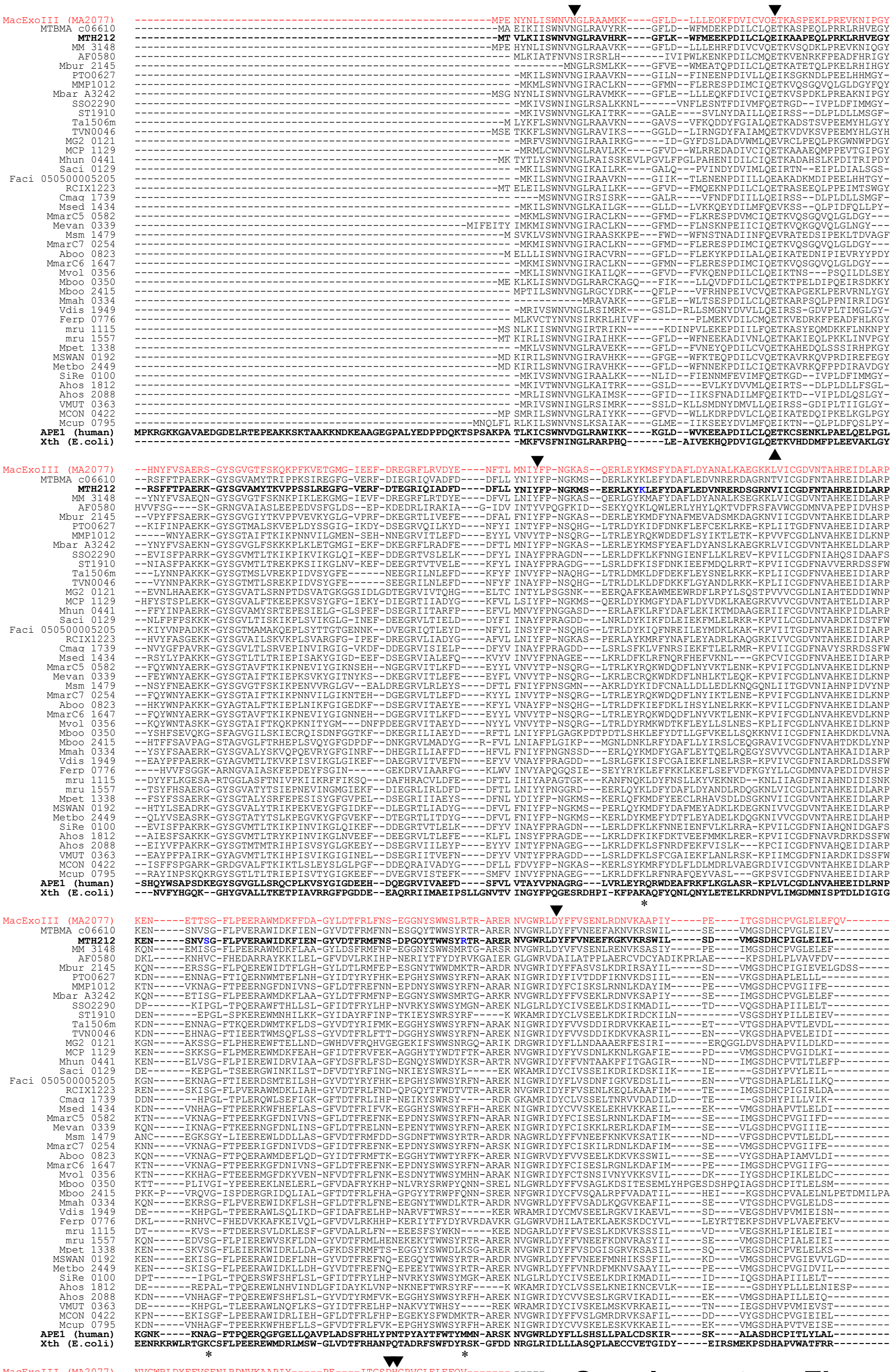

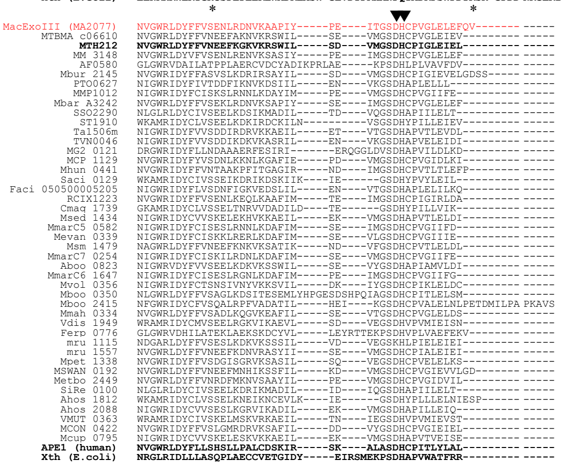

Supplementary Figure S1. Amino acid sequence alignment of ExoIII homologs. The 46 full-length amino acid sequences of archaeal homologs were aligned with human APE1, and *E. coli* Xth. The sequences from *E. coli*, *M. thermautotrophicus*, and *H. sapiens* are indicated with bold letters. MacExoIII is indicated with red letters. Catalytic residues are indicated with ▼ at the top. The putative residues in Mth212 contributing to the recognition of dU in DNA are indicated with cyan letters and their positions are indicated by asterisks at the bottom. ▲ indicates the mutated glutamic acid residue in this study (E39 in MacExoIII).

```
                                                                    ▼  ▼
MacUDG_MA_RS18745             MKELEIVAAEEENCGNFEERVQKLVEAGYETVAREAIACTRCPLHKSATKRVIGKGSCNP
MacUDGlike_MA_RS11760                                  MKPVEYVKCKAFPCSDINKGGYLVPAVEVNPEKVK
Sulfolobus solfataricus                                MDNLDLIADEVRKCQKCKLWKFRKNAVPGEGNSKA
Sulfolobus tokodaii                                    MDSLEKIKEEVISCKKCKLWQFRTNAVPGEGYPKA
Pyrococcus furiosus                                 MSKHELMKKLEEKILTCKKCPLWRLRTNPVPGYGNYDA
Archaeoglobus fulgidus                                 MESLDDIVREIMSCRKCDLHKTKTNYVPGVGNEKA
Aeropyrum pernix                                     MAGSRLRQLEEEVRRCTRCPLHATRTHAVPGEGPGEA
Pyrobaculum aerophilum                                  MDLQKLHELIKNCDKCPLHKYRKNAVPGEGEMKL
Thermus thermophilus                                    MTLELLQAQAQNCTACRLMEGRTRVVFGEGNPDA
Deinococcus radiodurans            MTGSLPPPRHPLHQAADPPDPAALLALEDRNRGCAACPLRVSASQVVVSDGDPRA
Thermotoga maritima                                  MYTREELMEIVSERVKKCTACPLHLNRTNVVVGEGNLDT
                                                          :         *            *

                                 Motif A                                               ▼
MacUDG_MA_RS18745             KVMFIGEAPGKTED---ETGIPFNG----RAGKKLDKMVEYMG-LSEEDWFVANTVKCRP
MacUDGlike_MA_RS11760         VVMIAEAPPENPLDYFYASGEPFYLKTTLQAFNDAGIPVNSIQEILDHGFYLTTALKCAK
Sulfolobus solfataricus       EIMFIGEAPGENED---IEGKPFVG----VAGKLLTRLINEILGLSREDVFITNLVKCRP
Sulfolobus tokodaii           EIMFVGEAPGENED---KEGRPFVG----AAGKLLTQMIKEILGLERDQVFITNVVKCRP
Pyrococcus furiosus           KIMFIGEAPGYWED---QKGLPFVG----KAGKVLDELLDGIG-LTREDVYITNVVKCRP
Archaeoglobus fulgidus        EIVFVGEAPGRDED---LKGEPFVG----AAGKLLTEMLASIG-LRREDVYITNVLKCRP
Aeropyrum pernix              GVMVVGEAPGRMED---RLGRPFVG----PAGKLLDSLLELAG-LSRGEVYITNVVKCRP
Pyrobaculum aerophilum        GVMIVGEAPGASED---EAGRPFVG----AAGQLLTEALSRLG-VRRGDVFITNVVKCRP
Thermus thermophilus          KLMIVGEGPGEEED---KTGRPFVG----KAGQLLNRILEAAG-IPREEVYITNIVKCRP
Deinococcus radiodurans       PLLIVGEGPGAEED---RDGRPFVG----QAGQLLDRILAAAS-LAREEAYLTNVTKCRA
Thermotoga maritima           RIVFVGEGPGEEED---KTGRPFVG----RAGMLLTELLRESG-IRREDVYICNVVKCRP
                               ::.    *    *     * **       *       :     :     :: .  **

                                           ▼
MacUDG_MA_RS18745             PENRRPKASEIECCK-PFLIAQITLLDPEIIILLGNTAEKAYCPERKLERGVPVEYEGR-
MacUDGlike_MA_RS11760         TR-YVISADTVKNCSLVLEKELSLFPNVEVYMLMGDVAIKAFNYISRRLTGKNTIRSGST
Sulfolobus solfataricus       PNNRDPEEDEILACS-PYLTRQIESIRPHIIITLGRHSTSYLFKKMNMKMESIGKVRGKF
Sulfolobus tokodaii           PNNRDPEEDEITACS-PYLDRQIDIIMPKIIVTLGRHSTKYIFSKMGENFSSITKVRGKS
Pyrococcus furiosus           PNNRDPTEEEIKACS-PYLDQQIDIIKPKVIVTLGRHSTNYILKKFGFDLEPISKIHGKV
Archaeoglobus fulgidus        PNNRDPTPEEVEKCG-DYLVRQLEAIRPNVIVCLGRFAAQFIFNLFDLEFTTISRVKGKV
Aeropyrum pernix              PGNRDPREEEIEACL-PYLVEQISLIRPRLVIAVGRHAGRTLFRLAGLRWPGLARARGRV
Pyrobaculum aerophilum        PNNRTPNREEVEACL-PYLIQQIGILKPRRIIALGLISAKALMELMGRRAEKLGDVKGKC
Thermus thermophilus          PQNRAPLPDEAKICTDKWLLKQIELIAPQIIVPLGAVAAEFFLG----EKVSITKVRGKW
Deinococcus radiodurans       PNNRTPLPLETATCTGLWLEPQLALLRPRVVLSLGNTATQFLLG----TPRGITRLRGQW
Thermotoga maritima           PNNRTPTPEEQAACG-HFLLAQIEIINPDVIVALGATALSFFVDG---KKVSITKVRGNP
MacUDG_MA3593                 PENRRPKASEIECCK-PFLIAQITLLDPEIIILLGNTAEKAYCPERKLERGVPVEYEGR-
MacUDGlike_MA2265             TR-YVISADTVKNCSLVLEKELSLFPNVEVYMLMGDVAIKAFNYISRRLTGKNTIRSGST
                              .            *                 : :*  :                   *

                                              Motif B
MacUDG_MA_RS18745             ------------MILKLYHPAALIYTASKIDVQKAFIDKNRDLWQ
MacUDGlike_MA_RS11760         YKIRKEQ---FFYNGKRVFPSYVQTGQNYLIEKSKRMMIAEDLAEAAKLIK
Sulfolobus solfataricus       YTWNIYG--YKILVFPTYHPAAALYNPPIRKVLEEDFRKVKEALSS-KPITLDNFLYG--
Sulfolobus tokodaii           YVWKYKE--KEIIVFPTYHPAAALYNPNLRKILEEDFKKIRELAITPKRYTIDYFLGGKN
Pyrococcus furiosus           FKAKTLF--GTLYIFPTYHPAVALYRPQLKEELKQDFDILKSLLEKLGI
Archaeoglobus fulgidus        YEVERWG--KKVKVIAIYHPAAVLYRPQLREEYESDFKKIGELCGKKQPTLFDYL
Aeropyrum pernix              WRGRIGG--VELLIAVTYHPAAALYNPGLRGELERDFSGFIRRSVAEALSRGGGGGGAGL
Pyrobaculum aerophilum        YQGRIAG--VQVELCITYHPAAVLRKPALRGEFQKDLAMFFG---------------GGL
Thermus thermophilus          YEWHG------IKVFPMFHPAYLLRNPSRAPGSPKHLTWLDIQEVKRALDALPPKERRPV
Deinococcus radiodurans       FTYRHPAWPQPALLMPLLHPAYLLRNPVRTPGGPKSLTWRDIREVAAVLRGEKEASPVQG
Thermotoga maritima           IDWLGG-----KKVIPTFHPSYLLRNRSNE---LRRIVLEDIEKAKSFIKKEG
                                                .*:               :

MacUDG_MA_RS18745
MacUDGlike_MA_RS11760
Sulfolobus solfataricus       -SGDKGEKGNSNSGK
Sulfolobus tokodaii           RSWDKREKSDSNSGK
Pyrococcus furiosus
Archaeoglobus fulgidus
Aeropyrum pernix              DRWFSPDSRGPGEGAGGDVDS
Pyrobaculum aerophilum        DRFLDPSK
Thermus thermophilus          KAVSQEPLF
Deinococcus radiodurans       QFPAAPDSLFSELE
Thermotoga maritima
```

Supplementary Figure S2. Amino acid sequence alignment of the characterized family-4 UDG proteins, MacUDG, and MacUDG-like (MA_RS18745 and MA_RS11760). Motif A: GE(A/G)PG, Motif B: HPAAVL, Underlined peptides: (putative or characterized) PIP box/β-clamp motif, ▾: Cysteine residues involved in Fe-S clusters. Accession numbers: *Sulfolobus solfataricus*, WP_009991844.1; *Sulfolobus tokodaii*, WP_010980322.1; *Pyrococcus furiosus*, WP_011012532.1; *Archaeoglobus fulgidus*, WP_010879766.1; *Aeropyrum pernix*, BAA79385.2; *Pyrobaculum aerophilum*, AAL62921.1; *Thermus thermophilus*, WP_011228142.1; *Deinococcus radiodurans*, WP_010888386.1; *Thermotoga maritima*, WP_004081422.1.

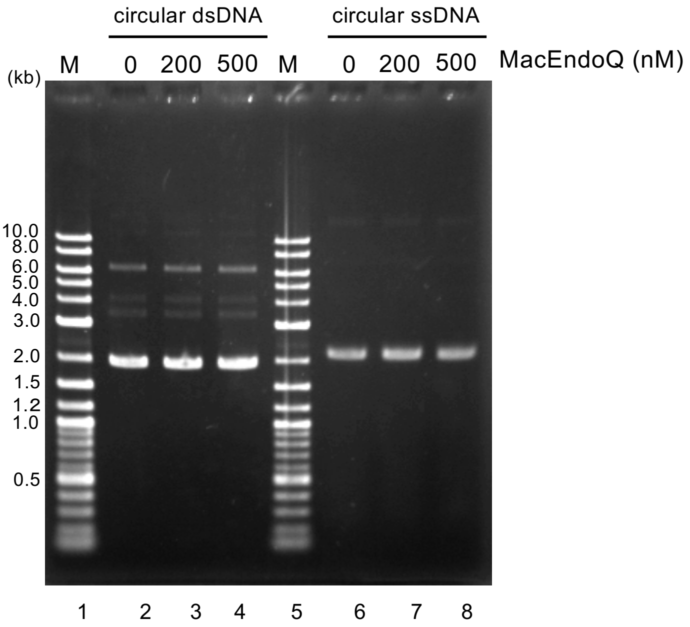

Supplementary Figure S3. Quality check of MacEndoQ sample. MacEndoQ (200 nM and 500 nM) was incubated with 20 ng/μl of supercoiled pBlueScript II SK(+) dsDNA (lanes 2–4) and pBlueScript II SK(+) ssDNA (lanes 6–8) at 37° C for 60 min in the reaction solution. Reaction products were analyzed by 0.8% agarose gel electrophoresis, followed by ethidium bromide staining. M, DNA marker (NEB, #3200).

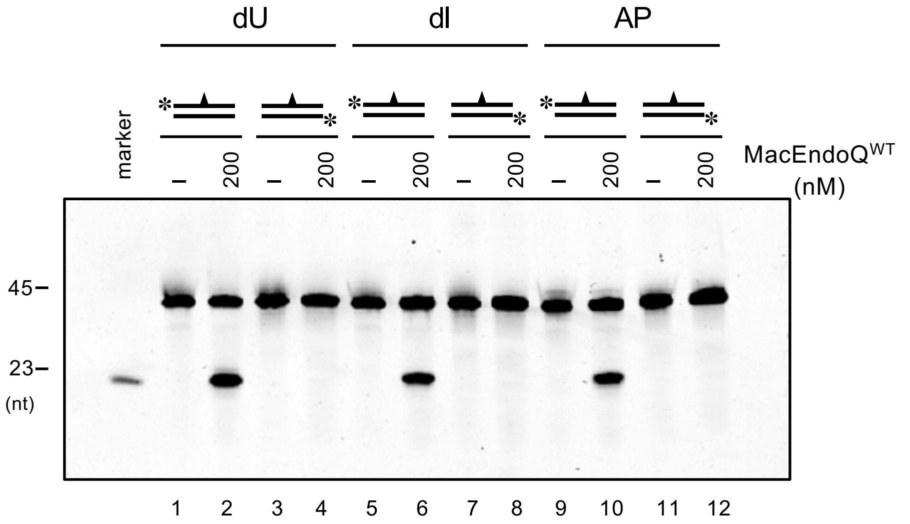

Supplementary Figure S4. Cleavage activity of MacEndoQ toward the opposite strand of a single lesion-containing strand. MacEndoQ cleavage reaction were performed at 37°C for 60 min using the single lesion-containing strand labeled DNA (lanes 1, 2, 5, 6, 9, 10) or the opposite (intact) strand labeled DNA (lanes 3, 4, 7, 8, 11, 12). For the detailed reaction conditions and the termination step, see the methods section in the main text. Cleavage products were separated by 8 M urea-12% PAGE.

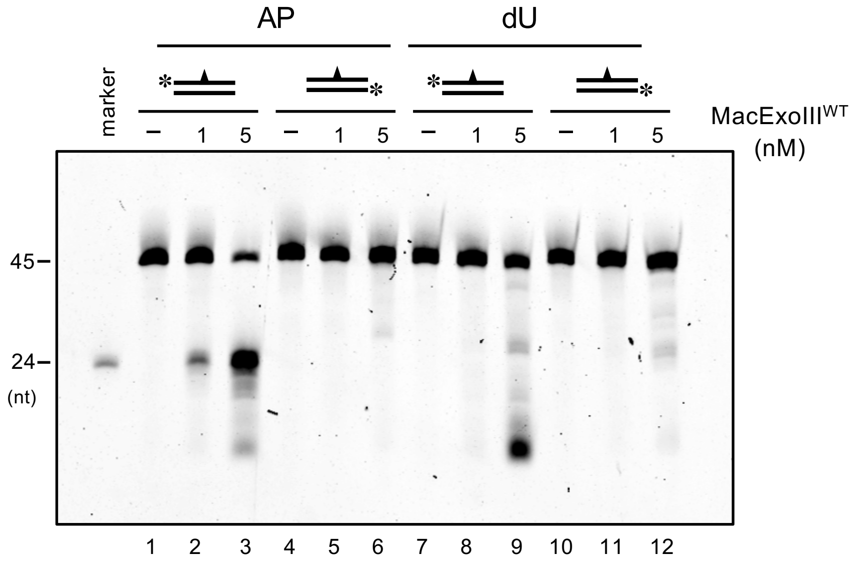

Supplementary Figure S5. Cleavage activity of MacExoIII toward the opposite strand of a single lesion-containing strand. MacExoIII cleavage reaction were performed at 37°C for 10 min using the (single lesion-containing) top strand labeled, blunt ended DNA (lanes 1–3, 7–9) or the bottom strand labeled, blunt ended DNA (lanes 4–6, 10–12). For the detailed reaction conditions and the termination step, see the methods section in the main text. Cleavage products were separated by 8 M urea-12% PAGE.

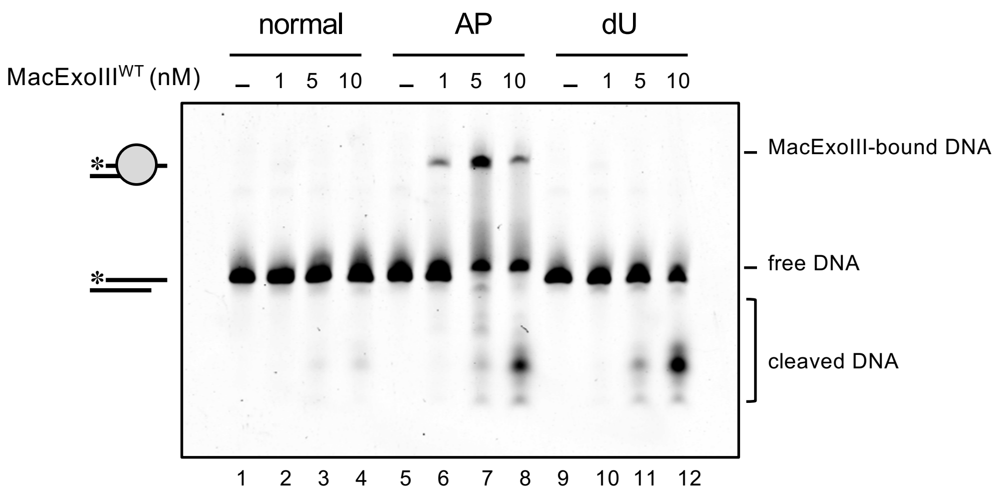

Supplementary Figure S6. Electrophoretic mobility shift assay of MacExoIII with normal/(AP/dU)-containing DNA. Various concentrations (0, 1, 5, and 10 nM) of MacExoIII^{WT} were incubated with 5 nM 5′-Cy5-labeled 6-nt 3′-overhang dsDNA (normal: lanes 1–4; AP site: lanes 5–8; dU: lanes 9–12), in reaction solution (50 mM Bis-Tris-HCl, pH 7.0, 1 mM DTT, 1 mM $MnCl_2$, 0.01% Tween 20; 20 µl) at 37°C for 10 min. After 5 µl of loading buffer (17% Ficoll, 10 mM Tris-HCl, pH 8.0 and 0.1% Orange G) was added, the samples were separated by 8% PAGE in TBE buffer and visualized by an image analyzer, Typhoon Trio+ (GE Healthcare). Assignments of the bands are shown on the left od the panel.

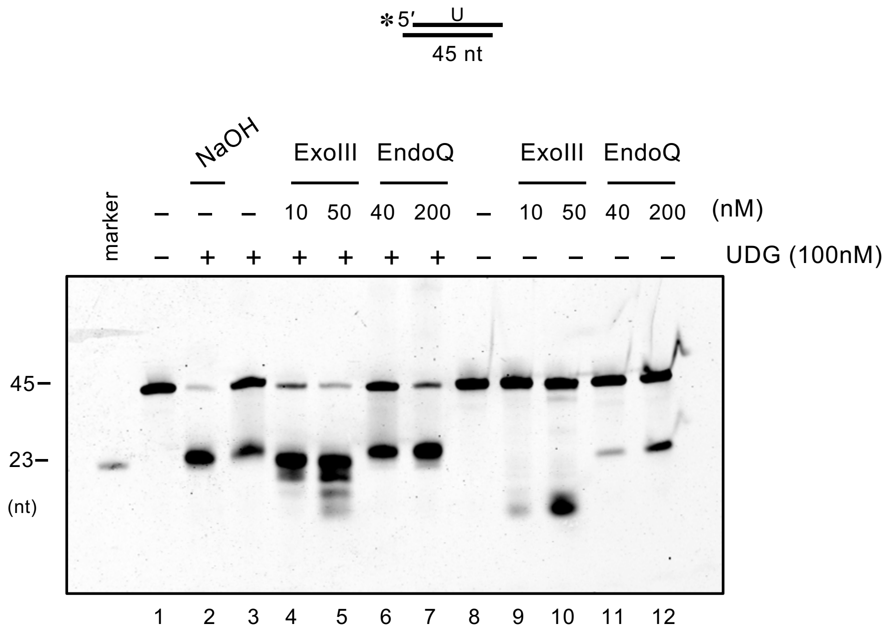

Supplementary Figure S7. Endonuclease activities by MacExoIII and MacExoQ toward MacUDG-catalyzed substrates. 5′-Cy5-labeled 6-nt 3′-overhanging, uracil-containing dsDNA were incubated with (100 nM; lanes 2–7) or without (lanes 1, 8–12) MacUDG in the 20 μl of reaction mixture (50 mM Tris-HCl, pH 8.0, 1 mM DTT, 0.1 μg/mL BSA) at 37°C for 10 min. After adding 1 μl of 20 mM $MnCl_2$ (lanes 1–5 and 8–10) or $MgCl_2$ (6, 7, 11, and 12), the indicated concentrations of MacExoIII^{WT} (lanes 4, 5, 9, 10) or MacEndoQ^{WT} (lanes 6, 7, 11, 12) (2 μl) were added to the reaction mixture and further incubated for 10 min. The lane 2 sample were treated with NaOH. For the details, see the methods section in the main text. Cleavage products were separated by 8 M urea-12% PAGE.

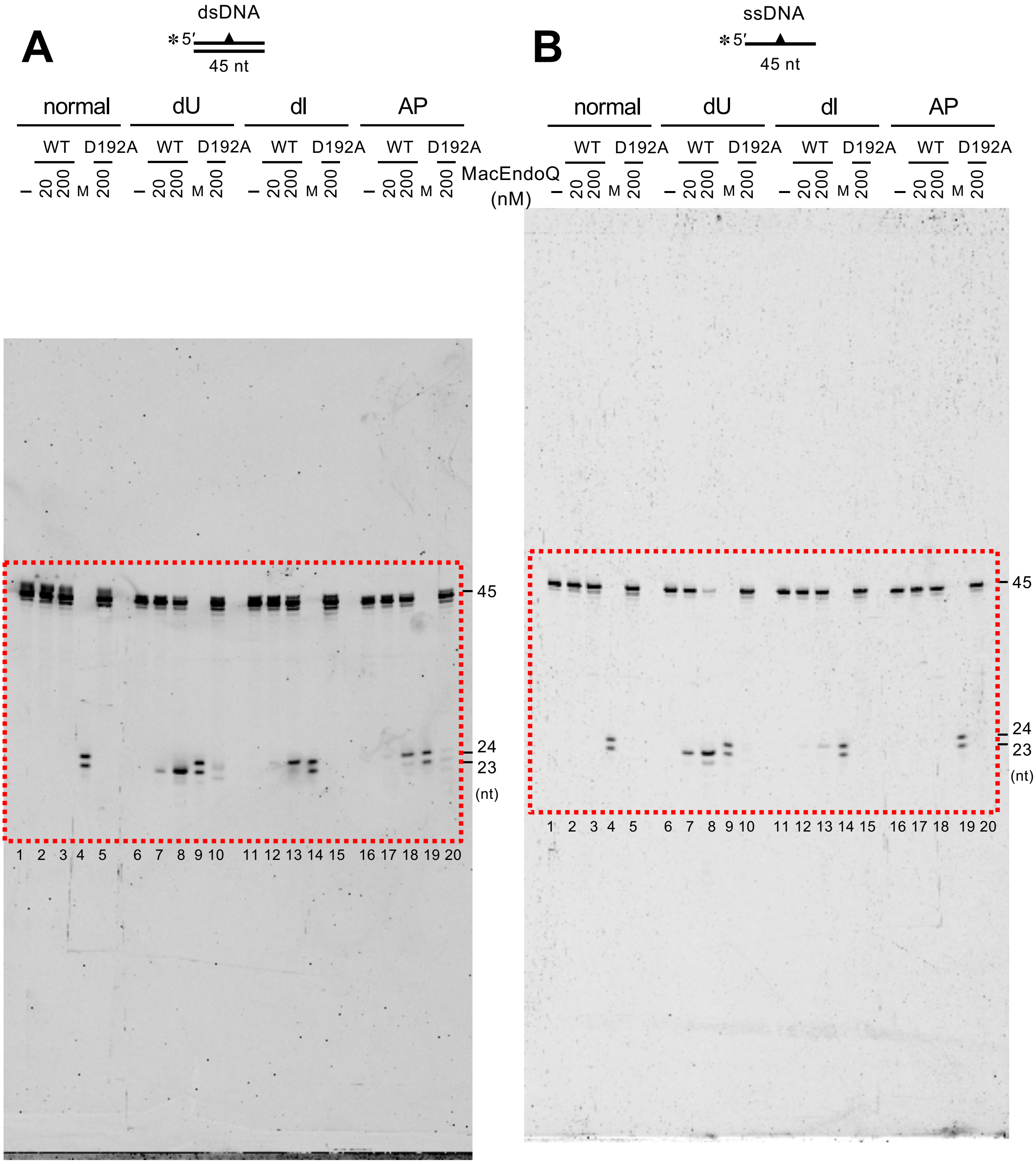

Supplementary Figure S8. Original gel images presented in Figure 2. Red dotted lines indicate the presented area in the main figure.

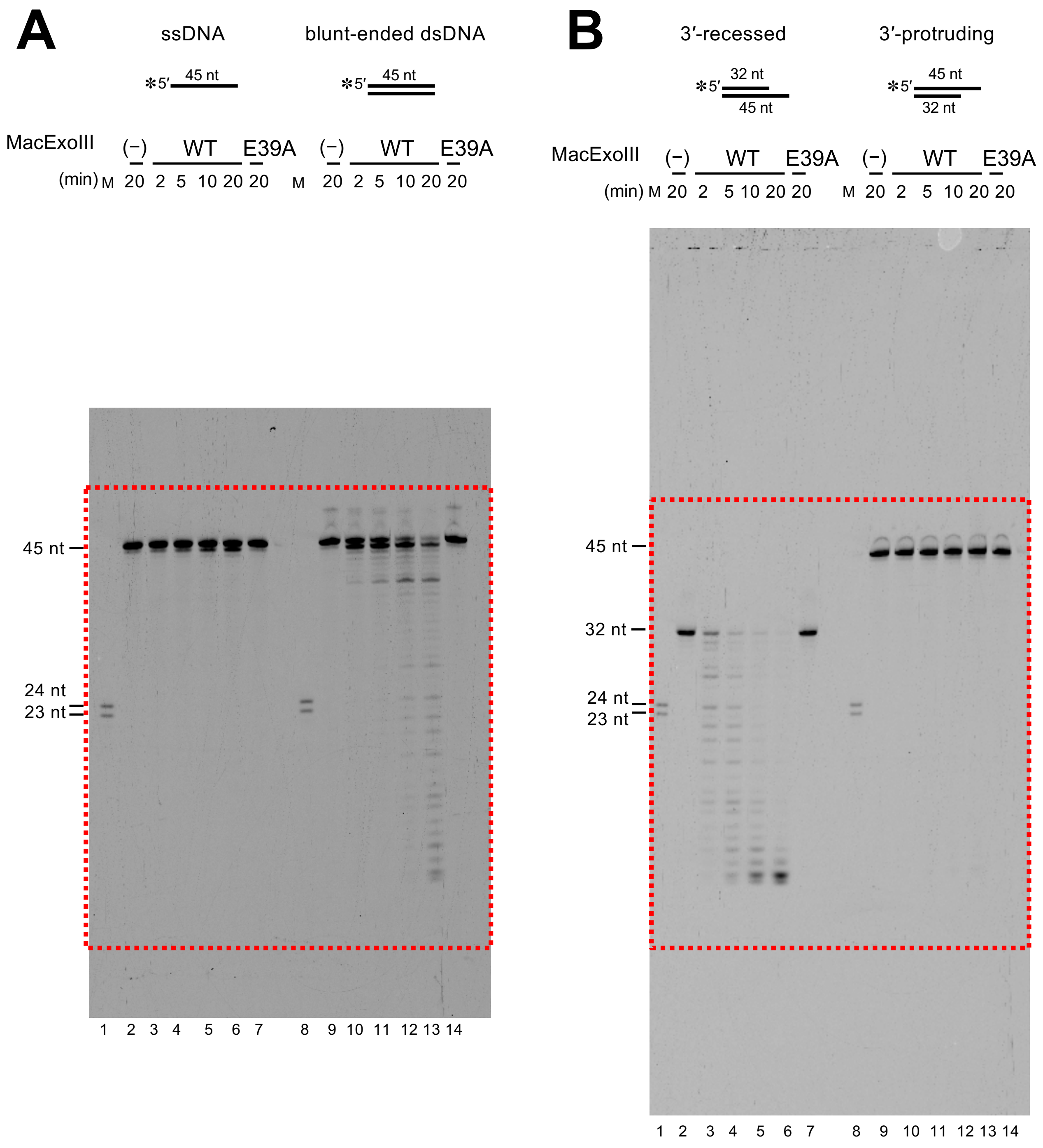

Supplementary Figure S9. Original gel images presented in Figure 3. Red dotted lines indicate the presented area in the main figure.

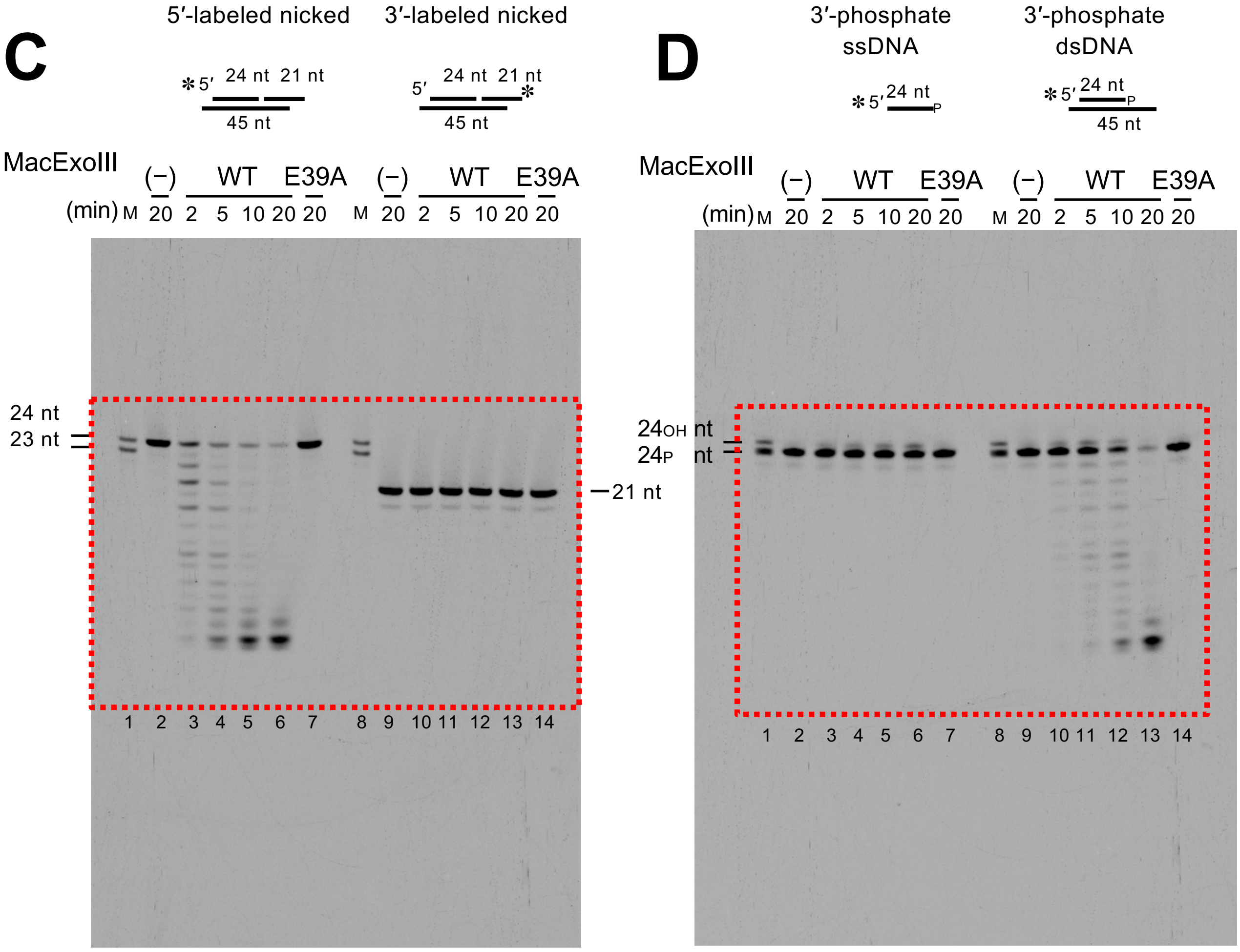

Supplementary Figure S9 (Continued). Original gel images presented in Figure 3. Red dotted lines indicate the presented area in the main figure.

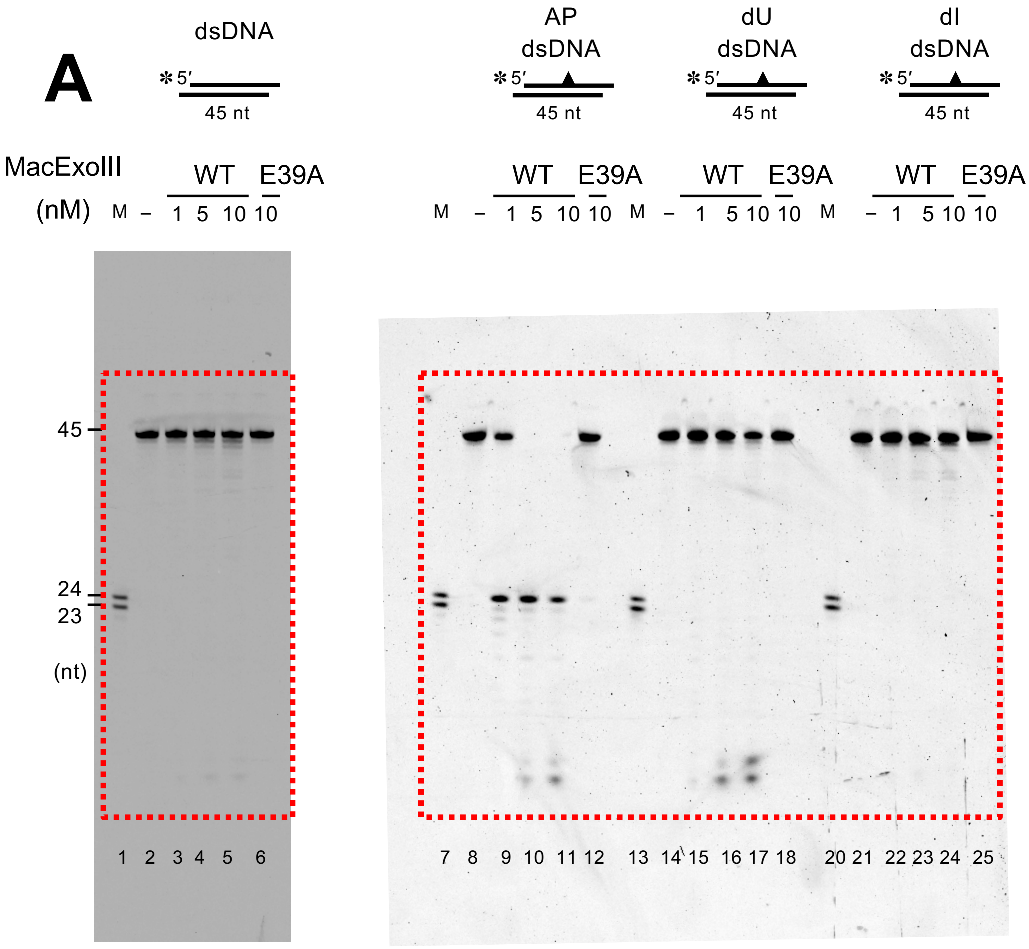

Supplementary Figure S10. Original gel images presented in Figure 4. Red dotted lines indicate the presented area in the main figure.

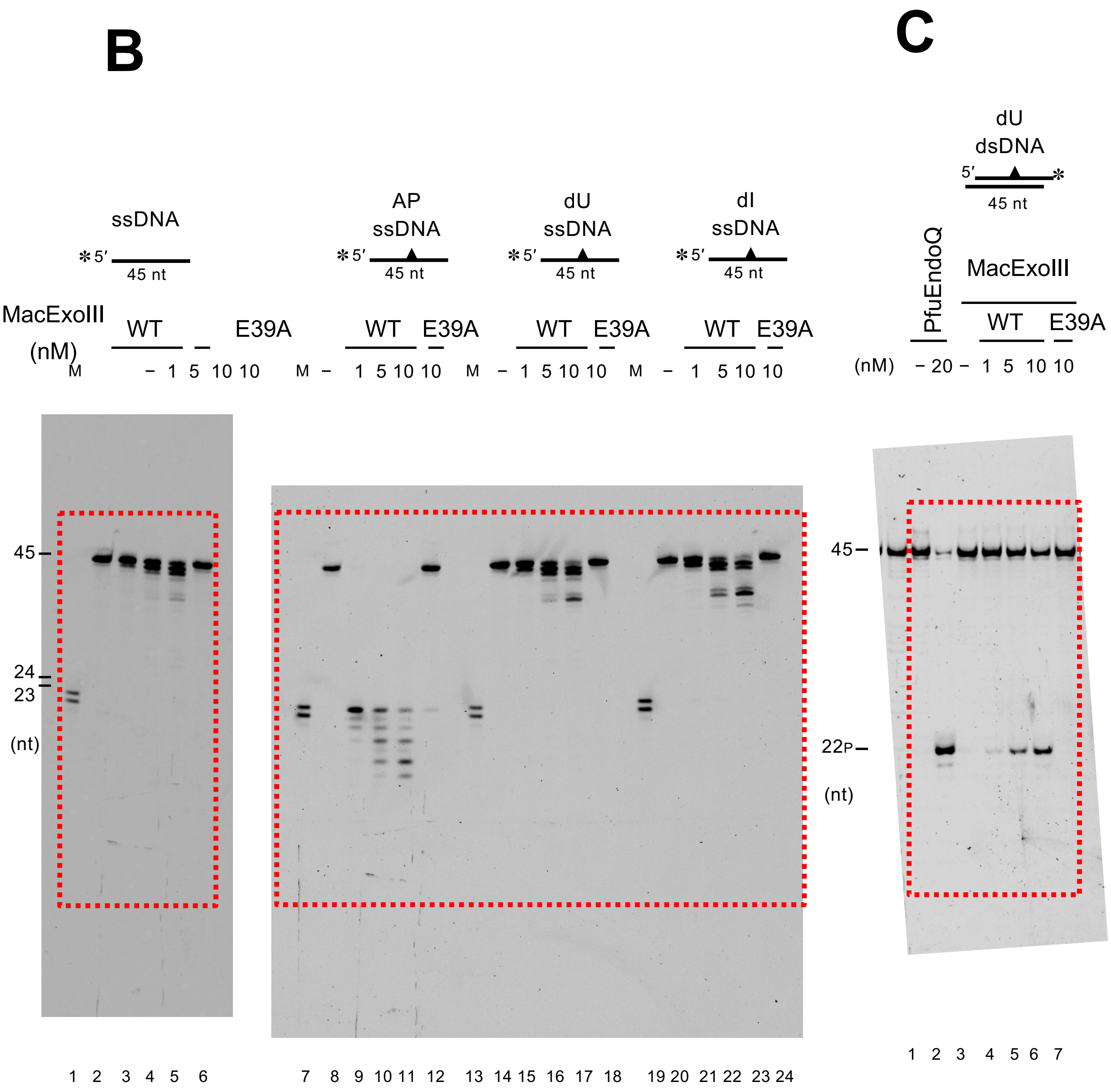

Supplementary Figure S10 (Continued). Original gel images presented in Figure 4. Red dotted lines indicate the presented area in the main figure.

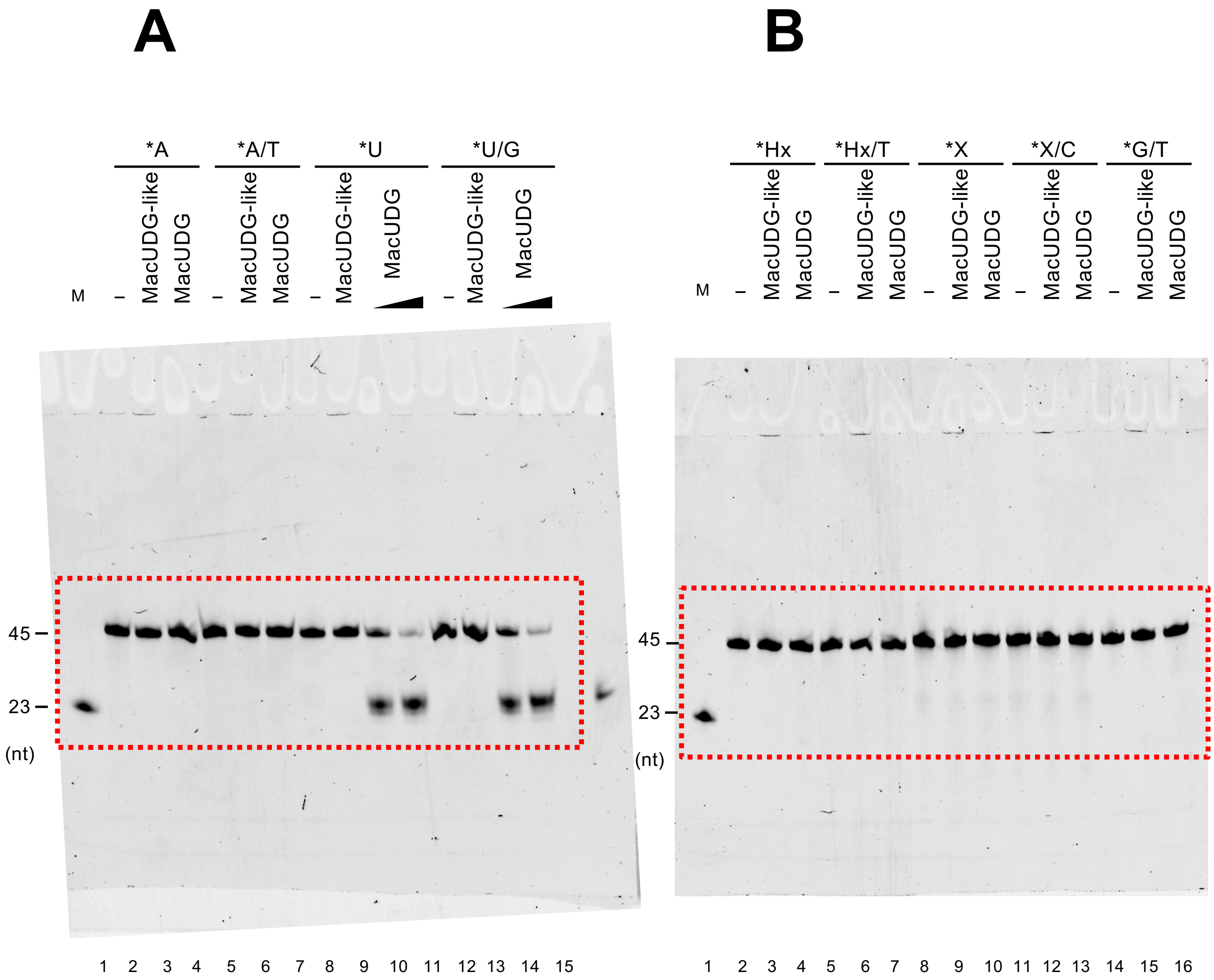

Supplementary Figure S11. Original gel images presented in Figure 5. Red dotted lines indicate the presented area in the main figure.
